# Lacritin cleavage-potentiated targeting of iron - respiratory reciprocity promotes bacterial death

**DOI:** 10.1016/j.jbc.2025.108455

**Published:** 2025-03-26

**Authors:** Mohammad Sharifian Gh., Fatemeh Norouzi, Mirco Sorci, Tanweer S. Zaidi, Gerald B. Pier, Alecia Achimovich, George M. Ongwae, Binyong Liang, Margaret Ryan, Michael Lemke, Georges Belfort, Mihaela Gadjeva, Andreas Gahlmann, Marcos M. Pires, Henrietta Venter, Thurl E. Harris, Gordon W. Laurie

**Affiliations:** 1 Department of Cell Biology, University of Virginia, Charlottesville, Virginia, USA; 2 Howard P. Isermann Department of Chemical and Biological Engineering and the Center for Biotechnology and Interdisciplinary Studies, Rensselaer Polytechnic Institute, Troy, New York, USA; 3 Division of Infectious Disease, Department of Medicine, Brigham and Women's Hospital, Harvard Medical School, Boston, Massachusetts, USA; 4 Department of Chemistry, University of Virginia, Charlottesville, Virginia, USA; 5 Department of Physiology, University of Virginia, Charlottesville, Virginia, USA; 6 Department of Pharmacology, University of Virginia, Charlottesville, Virginia, USA; 7 Sansom Institute for Health Research, University of South Australia, Adelaide, Australia; 8 Department of Biomedical Engineering, University of Virginia, Charlottesville, Virginia, USA; 9 Department of Ophthalmology, University of Virginia, Charlottesville, Virginia, USA

**Keywords:** lacritin, pathogen, respiration, FeoB, PotH, transporter, receptor

## Abstract

Discovering new bacterial signaling pathways offers unique antibiotic strategies. With current antibiotic classes targeting cell wall synthesis, depolarizing the inner membrane, altering the bacterial metabolome or inhibiting replication or transcription pathways, manipulation of transporters to limit bacterial respiration and thereby pathogenesis has been a decades-long quest. Here we report an inhibitor of multiple bacterial transporters. The inhibitor is the bactericidal N-104 endogenous cleavage fragment of the prosecretory mitogen lacritin. Lacritin is now known to be widely distributed in plasma, cerebral spinal fluid, tears, and saliva. With the bactericidal mechanism determined to be nonlytic by surface plasmon resonance as confirmed by lack of SYTOX Orange entry, we performed an unbiased resistance screen of 3884 *Escherichia coli* gene knockout strains revealing a complex N-104 polypharmacology. Validation in the virulent *Pseudomonas aeruginosa* strain PA14—one of three WHO Priority 1: Critical list species—focused on an approach that sequentially couples three transporters and downstream transcription to lethally suppress respiration. By targeting the outer membrane YaiW, cationic N-104 translocates into the periplasm where it ligates inner membrane transporters FeoB and PotH, respectively, to suppress both ferrous iron and polyamine uptake. With FeoB favoring an anaerobic environment, N-104 promotes the expression of genes regulating anaerobic respiration while largely suppressing those involved in aerobic respiration—a strategy counterproductive under aerobic conditions. This mechanism is innate to the surface of the eye and is enhanced by synergistic coupling with tear thrombin fragment GKY20 as tested on antibiotic-resistant clinical isolates.

Prokaryotic respiration is largely the responsibility of evolutionarily ancient multisubunit transporters in their outer and/or inner membranes (1). Manipulation of transporters to limit bacterial respiration, and thereby pathogenesis, has been a decades-long quest—particularly those who competitively acquire extracellular iron essential for energy production, oxygen transport, gene regulation, and virulence (2). Other targets involve polyamine uptake for growth, biofilm formation, swarming, and siderophore synthesis (3), and transporters capable of antibiotic expulsion that in turn can enhance resistance. Yet, among current preclinical anti-bacterial approaches few directly address transporters. None directly inhibit more than one transporter. In contrast, current antibiotic classes target cell wall synthesis, depolarize the inner membrane, alter the bacterial metabolome, or inhibit replication or transcription pathways. Many obstruct translational pathways.

Identifying an inhibitor of multiple transporters might be transformational, as virulent pathogens have evolved many elegant ways to overcome host nutritional immunity. Direct uptake of the neutral pH soluble Fe^2+^ (ferrous) form of iron occurs mainly by the anaerobically induced transporter FeoB under conditions of hypoxia when Fe^2+^ is the most common form of iron (4). Its availability enhances the virulence of the opportunistic pathogen *Pseudomonas aeruginosa* in individuals with cystic fibrosis (5). FeoB is widely represented in the inner membrane of Gram-negative bacteria (including *Enterobacteriaceae*, *Acinetobacter*, and *Pseudomonas*) and the sole membrane of Gram-positive bacteria (including *Staphylococci*) and in archaea, with modeling suggesting a GTP gated pore structure (6). Unlike Fe^2+^, the oxidized Fe^3+^ (ferric) form of iron is insoluble at neutral pH, although it is more abundant and the primary iron source when the host environment is normoxic. Bacterial uptake is indirect through secreted chelating siderophores that displace host Fe^3+^ sequestered on ferritin, lactoferrin and transferrin for capture by bacterial surface receptors and transport by the TonB:ExbB:ExbD and lipoprotein siderophore-binding protein systems on Gram-negative and Gram-positive bacteria, respectively.

Out of an unbiased biochemical screen for exocrine secretagogues, we previously identified (7) and characterized the homeostasis restorative (8, 9, 10), autophagy (11), regenerative (12), and secretory (7, 13, 14) agonist lacritin whose cleavage-potentiated release of the cationic C-terminal N-104 15-mer fragment protects the eye's wet mucosal surface from environmental pathogens (15, 16). The nature of this bactericidal activity is largely unknown, although metabolomic analysis of treated *Escherichia coli* (15) suggested a metabolic stress-killing mechanism. Here, we discover that N-104 acts *via* a complex polypharmacology. We focus on its role as a direct inhibitor of the Fe^2+^ transporter FeoB and polyamine transporter subunit PotH in *E. coli* and in the virulent, multidrug-resistant *P. aeruginosa* strain PA14. Iron and polyamines are essential for bacterial survival. With FeoB favoring an anaerobic environment, N-104 promotes the expression of genes regulating anaerobic respiration while largely suppressing those involved in aerobic respiration—a strategy counterproductive under aerobic conditions. N-104's activity is enhanced by synergistic coupling with the tear and plasma thrombin peptide GKY20. Thus, lacritin promotes host homeostasis in multiple ways.

## Results

### Killing without lysis

**Figure 1:**
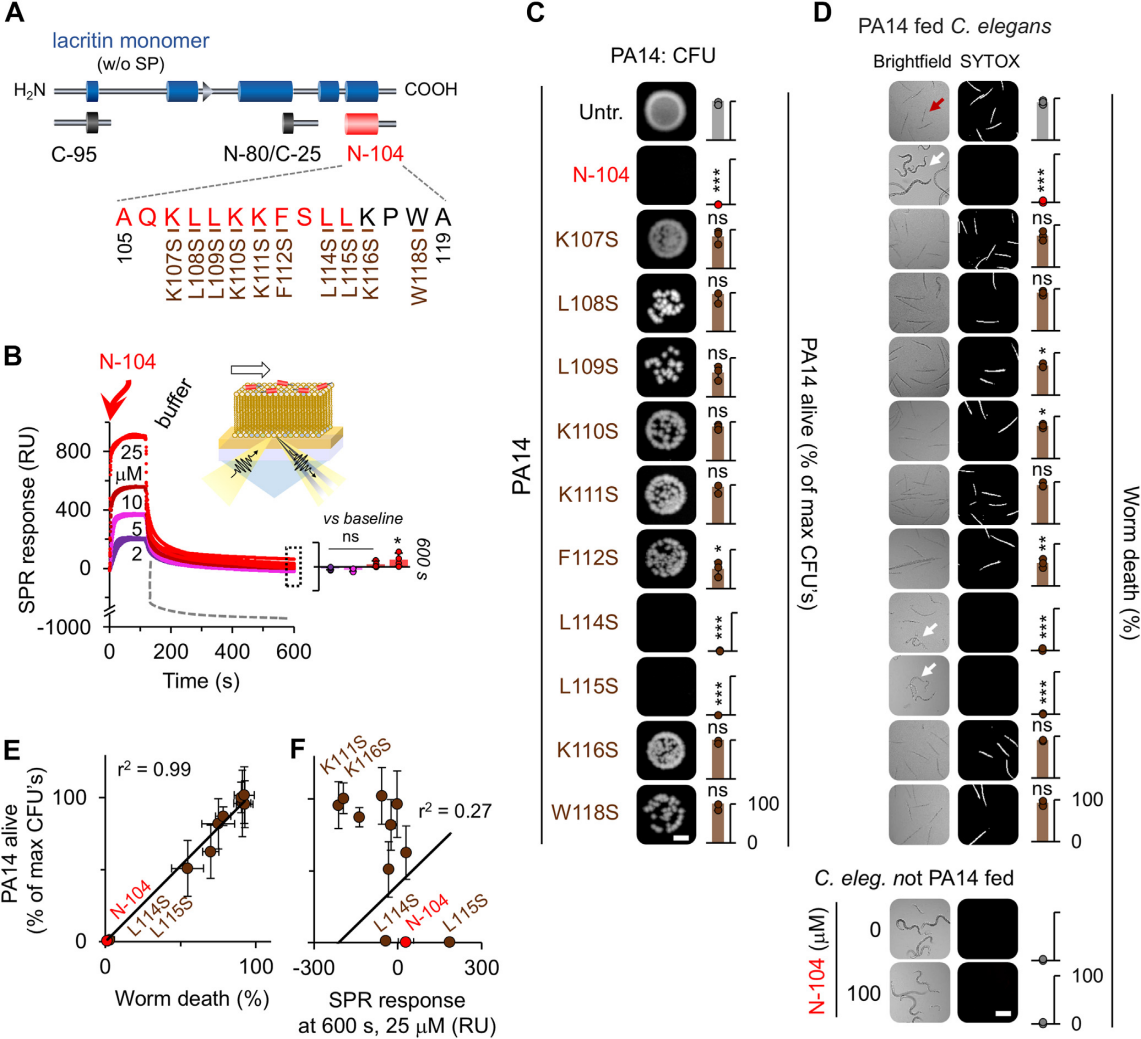
**Lysis-free N-104 killing of virulent and multidrug-resistant *P. aeruginosa* strain PA14.***A*, linear diagram of secreted lacritin monomer with blue rectangles indicating confirmed (C-terminal half) or PSIPRED predicted α-helices and a β-strand (*arrowhead*). Release of bactericidal proteoform N-104 (here using synthetic peptide as a surrogate) is by proteolysis (10, 15). The synthetic peptides C-95 and N-80/C-25 lack the N-104 bactericidal domain and thus serve as negative controls. Synthetic N-104 analogs with serine replacing an amino acid with nonpolar or basic side chains are shown (*brown font*). Lacritin is a tear, saliva, plasma, and cerebral spinal fluid glycoprotein. *B*, surface plasmon resonance response (SPR) following flow (30 μl/min) of 2, 5, 10 or 25 μM N-104 (*red arrow*) over chip-supported PC:PG:PE ((59:21:20 (20); schematic inset) followed at 150 s by buffer wash at the same flow rate. The dashed gray line indicates the precipitous SPR collapse when a lytic peptide dislodges lipid (22). The dotted box indicates 600 s data enlarged in the *right inset* ([mean with S.D.] n = 4, data *versus* baseline ∗*p* < 0.05; ns, not significant [two-way ANOVA with Tukey's multiple comparison test]). *C*, *P. aeruginosa* (PA14) colony forming unit (CFU) assay whereby overnight culture of PA14 (10^6^ cfu/ml) was incubated in suspension for 8 h without (untreated; Untr) or with 25 μM N-104 or each N-104 analog and then plated onto LB agar. The scale bar is 2 mm. *Right*, quantitation ([mean with S.D.], n = 3, ∗∗∗*p* < 0.001; ∗*p* < 0.05; ns, not significant [unpaired *t* test]; quantitation was *versus* W118S; W118S analysis was *versus* K116S). *D*, *C. elegans* survival assay in which overnight cultures of PA14 (10^7^ cfu/ml) either untreated or treated for 5 h with 100 μM N-104 (or with 100 μM of each N-104 analog) were fed to 50 *C. elegans* worms per well in 96-well plates for 3 days. Rather than PA14, some were fed for 3 days without and with 100 μM N-104. After the removal of bacteria, an equal volume of SYTOX Orange was added to fluorescently image dead worms. The same approach was employed for all other *C. elegans* experiments. Scale bar is 200 μm. *Right*, quantitation ([mean with S.D.], n = 3, ∗∗∗*p* < 0.001; ∗∗*p* < 0.01; ∗*p* < 0.05; ns, not significant [unpaired *t* test]; quantitation was *versus* W118S; W118S analysis was *versus* K116S). *E*, linear regression of PA14 viability *versus* worm death ([mean with S.D.] n = 3). *F*, Linear regression of PA14 viability *versus* the SPR response at 600 s of 25 μM N-104 or N-104 analogs ([mean with S.D.] n = 3).

Innate protection of the surface of the eye is dependent in part on protease-potentiated release of the 15 amino acid C-terminal N-104 cationic domain (Fig. 1*A*) (15) from the tear and salivary protein lacritin (17). Lacritin is now known to be also in plasma and cerebral spinal fluid. The bactericidal domain largely consists of an amphipathic α-helix (Fig. S1, *A* and *B*), but how it kills is unknown. Amphipathic cationic peptides are often bacteriolytic or form pores or disturb bacterial membranes in other ways (18). N-104 is contained within the 54 amino acid recombinant fragment N-65. N-65 allowed the entry of membrane-impermeable SYTOX Green into *E. coli*, although metabolomic changes were incompatible with lysis (15). Possibly N-65 may have created pores. Yet with much smaller N-104 at the elevated concentration of 25 μM, no SYTOX Orange uptake was observed in the multidrug-resistant *P. aeruginosa* PA14 strain (Fig. S1*E*). N-104 overlaps with the lacritin N-94/C-6 and N-94 proteoforms. Both interact with the (O-acyl)-ω-hydroxy fatty acid class of amphiphilic lipids in the eye (10). Could N-104 nonetheless be membrane interactive? Surface plasmon resonance (SPR) is a highly sensitive approach for detecting lipid loss as a consequence of strong cationic peptide affinity for chip-supported bilayer membranes. SPR sensitivity is as little as 10 pg/ml of dry mass (19). We formed chip-supported membranes of PC:PG:PE (Fig. 1*B*) and PC:PG (Fig. S1*F*), previously employed as simple approximations respectively of Gram-negative (20) and Gram-positive (21) bacterial membranes, although lacking many elements. The flow of N-104 over each gave rise to a peptide concentration-dependent increase in the SPR response, that however did not precipitously collapse (dashed line) with buffer wash (Figs. 1*B* and S1*F*). A precipitous SPR collapse is observed when peptide-disrupted membrane lipid is dislodged by the buffer wash (22). Instead, at the highest concentration of N-104, the SPR response remained slightly elevated over baseline (Fig. 1*B*) on the Gram-negative membrane in keeping with N-104 surface aggregation, or slightly below baseline (Fig. S1*F*; Gram-positive membrane). Thus, N-104 is not membrane lytic nor substantially membrane disruptive.

An alternative possibility is partial N-104 membrane translocation, thereby increasing the SPR-detected mass. Some nonlytic antimicrobial peptides utilize multiple tryptophans, or both arginines and prolines, or a single-helix disruptive proline to translocate bacterial outer and inner membranes (23). N-104 lacks arginine and contains only a single proline and tryptophan (Fig. 1*A*), but is enriched with four lysines and leucines as well as a single phenylalanine and alanine. Lysines can electrostatically interact with the negatively charged outer membrane. Leucine and phenylalanine can facilitate bacterial membrane insertion. To examine these possibilities, we synthesized 10 N-104 analogs, each with a single serine substitution for lysine, leucine, phenylalanine, and tryptophan. Functionally relevant changes may be reflected by killing potential in the context of the SPR response. We first challenged the multidrug-resistant *P. aeruginosa* PA14 strain with each analog or N-104 using a colony-forming unit assay (CFU). Only the L114S and L115S analogs retained full N-104 bactericidal activity (Fig. 1*C*) at a minimum inhibitory concentration (MIC) of 2 μM (3.6 μg/ml; Fig. S1*C*). We also fed each analog- or N-104-treated PA14 to *Caenorhabditis elegans*. The *C. elegans* survival assay is considered a model of mammalian pathogenic infection (24). Live PA14 kills *C. elegans*. Dead *C. elegans* is detected by SYTOX Orange. Only *C. elegans* fed PA14 treated with bactericidal L114S, L115S, and N-104 fully survived (Fig. 1*D*). Thus, by killing PA14 each was protective of *C. elegans* (Fig. 1*E*). In control experiments, feeding *C. elegans* with N-104 alone did not affect viability (Fig. 1*D*, bottom). Thus, all amino acids with basic side chains and most with nonpolar side chains contribute to the N-104-killing activity. Each analog was then subjected to SPR. Most analogs promoted a decrease in the SPR response, particularly K111S and K116S (Fig. 1*F*), however PA14 killing capacity of each was neutralized or largely diminished. One exception was L114S whose SPR response was slightly (Fig. 1*F*) below that of N-104. Only L115S substantially boosted the SPR response presumably either *via* surface aggregation or membrane translocation (Fig. 1*F*). L115S was equally bactericidal as N-104. No correlation was observed between the SPR response and PA14 killing (r^2^ = 0.27; Fig. 1*F*). The results were similar on the Gram-positive membrane. Yet, L114S was both membrane-disruptive and bactericidal (Fig. S1*F*). With this exception, we conclude that N-104 does not disrupt membranes and that N-104 surface aggregation or membrane translocation, particularly by L115S, may be involved.

### Lack of PA14 generational resistance to N-104

*P. aeruginosa* is endowed with a robust capacity to repel or develop resistance against cationic antibiotics through intrinsic low outer membrane permeability and the development of reduced uptake or enhanced efflux through mutation or altered gene expression (25). Resistance often develops by 10 generations (26), particularly when antibiotic exposure is below the minimal inhibitory concentration. To determine how well PA14 might develop resistance to N-104, we treated PA14 with N-104 through 30 successive generations at subminimal inhibitory and minimal inhibitory concentrations. No stable resistance was apparent (Fig. S2, *A*–*C*).

### N-104 resistance screen of 3884 single gene knockouts identifies candidate mediators

**Figure 2:**
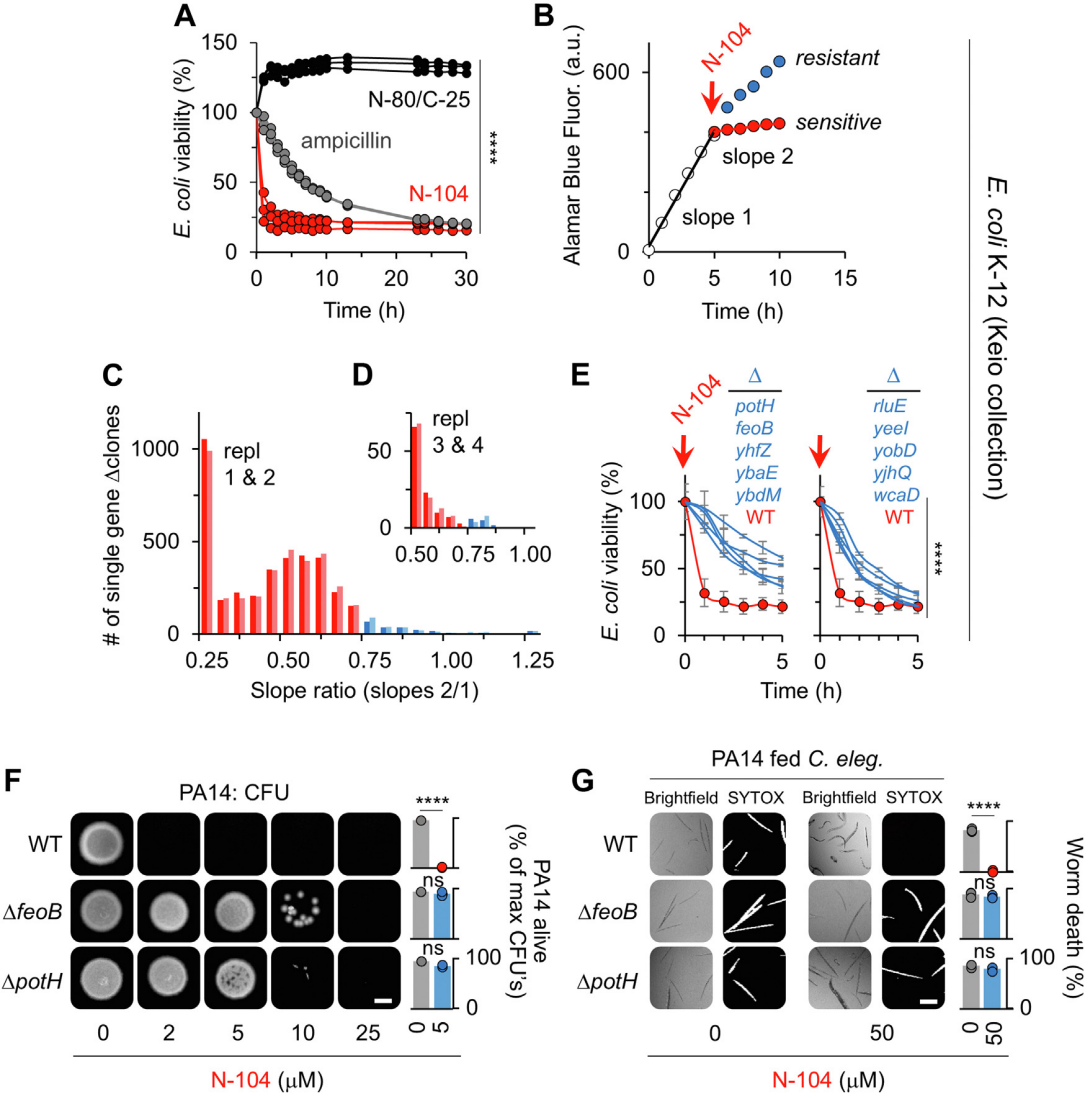
**Forward genetic N-104 resistance screen of the *E. coli* Keio collection of 3884 single gene knockouts identifies a putative polypharmacology of ten direct or indirect mediators.***A*, optimization assay in which overnight suspension cultures of wild-type *E. coli* K-12 (10^6^ cfu/ml) in wells of 96-well plates were treated at 37 °C with 100 μM N-104, N-80/C-25 (negative control) or ampicillin (positive control), or left untreated. Culture viability was monitored hourly with 10% Alamar Blue (87). Data is shown as the change over untreated relative to viability at T0 (n = 3, ∗∗∗∗*p* < 0.0001 [two-way ANOVA with Dunnett's multiple comparisons test]). *B*, schematic of mutant growth for 5 h before (slope 1) and after (slope 2) N-104 addition (*red arrow*). Hypothetical slope 2 of N-104-sensitive *versus* -resistant Keio collection mutants are respectively indicated by *red* and *blue-filled circles*. This approach was utilized in *C* and *D*. *C*, forward genetic resistance screen showing replicate slope ratios of each of 3884 Keio collection mutants (10^6^ cfu/ml) in 96 well plate suspension cultures after treatment at 5 h with 100 μM N-104. In controls, mutants at 5 h were treated with 100 μM N-80/C-25 and ampicillin (not shown). Mutants with a slope ratio ≥ 0.75 (*blue bars*) were considered to be N-104 resistant (n = 2). *D*, slope ratios of the 122 N-104 resistant mutants from *C* after replicate rescreening for N-104 resistance *C* (n = 2). *E*, Comparative viability over 5 h of 100 μM N-104 treated wild type *E. coli* K-12 and each of the 10 N-104 resistant single gene knockout mutants (each 10^6^ cfu/ml; [mean with S.D.], n = 3, ∗∗∗∗*p* < 0.0001 [two-way ANOVA with Šidák's multiple comparisons test]). *F*, overnight CFU assay of wild-type PA14 (WT), PA14 Δ*feoB*, and PA14 Δ*potH* transposon mutants (10^6^ cfu/ml) after treatment in suspension for 8 h with 0 to 25 μM N-104 and then plated onto LB agar. The scale bar is 2 mm. *Right*, quantitation ([mean with S.D.], n = 3, ∗∗∗∗*p* < 0.0001; ns, not significant [unpaired *t* test]). *G*, *C. elegans* survival assay in which overnight cultures of wild-type PA14, PA14 Δ*feoB*, and PA14 Δ*potH* (10^7^ cfu/ml) were incubated in suspension with 0 or 50 μM N-104 for 5 h and then fed to *C. elegans* as per Figure 1*D*. Scale bar is 200 μm. Quantitation at right ([mean with S.D.], n = 3, ∗∗∗∗*p* < 0.0001; ns, not significant [two-way ANOVA with Šidák's multiple comparisons test]).

To explore N-104’s killing mechanism, we screened each of the 3884 Keio *E. coli* K-12 collection (27) single gene knockout strains for N-104 resistance, aware of newly apparent transcriptional effects downstream of deleted genes (28). Others employed the same approach to identify the role of the CpxR/CpxA two-component system, and both EnvC and ZapBin, in *E. c*oli resistance respectively to the cationic antimicrobial peptides protamine (29) and human neutrophil peptide 1 (30). Our endpoint assay was bacterial metabolic activity as monitored through the reduction of resazurin in Alamar Blue. This facilitated high throughput screening in which N-104 and positive control ampicillin were inhibitory but not negative control N-80/C-25 (Fig. 2*A*). The N-80/C-25 synthetic peptide derives from an inactive region of lacritin (Fig. 1*A*). We then developed a screening strategy that compared the 5 h Alamar Blue detectable metabolic activity of each before (Fig. 2*B*, slope 1) and after (Fig. 2*B*, slope 2) N-104 treatment, recognizing that the metabolic activity of each knockout strain may differ. A treated:untreated metabolic slope ratio of 0.75 or greater (Fig. 2*B*; blue dots) was considered to be indicative of N-104 resistance. N-104 and negative and positive controls were applied at a relatively high 100 μM (180 μg/ml of N-104) dose to minimize ambiguous outcomes. Duplicate N-104 screens of the entire collection yielded 122 resistant candidates (Fig. 2*C*, small blue columns). These were further screened twice (Fig. 2*D*) thereby narrowing the list to 10 (Fig. 2*E*) - each with a Z-score ≥ 1.85. Such complex polypharmacology pointed to the possibility that N-104 may have more than one target or that some or all were functionally linked.

Gram-negative bacterial proteins are distributed throughout the outer and inner bilayer membranes, as well as in the ∼25 nm wide peptidoglycan-rich periplasm between and in the cytoplasm. Resistance was attributable to absence (Table 1) of the: (i) inner membrane ferrous iron transporter FeoB, (ii) inner membrane polyamine transporter subunit PotH (31), (iii) predicted inner membrane colanic acid polymerase WcaD (32) involved in biofilm formation, (iv) the growth-promoting antitoxin YjhQ of the YjhX-YjhQ toxin-antitoxin system (33), (v) zinc metallopeptidase MtfA (YeeI) (34), (vi) inner membrane DUF986 protein YobD (35); or to one of four regulatory factors: (vii) periplasmic SgrR family member YbaE (36), (viii) cytosolic ParB-like nuclease domain-containing YbdM (37), (ix) GntR family member RluE (38) (YmfC), or (x) putative transcription factor YhfZ (39). BLAST of each against the nonredundant protein sequence database (Table S1) revealed a common distribution among Enterobacteriaceae, and some also in *Acinetobacter baumannii* (FeoB, PotH, YobD, YbdM, RluE, YhfZ) and *P. aeruginosa* (FeoB, PotH, WcaD, YbdM, RluE)—all Gram-negative opportunistic pathogens. We chose to focus our efforts on FeoB and PotH since both are common to all three pathogen groups and together with RluE, a 23S rRNA pseudouridine synthase (Table S1), are the only three proteins expressed in multidrug-resistant *P. aeruginosa* PA14 of the ten. We tested PA14 transposon mutants (40) of FeoB and PotH in CFU (Figs. 2*F* and S2*D*) and *C. elegans* survival (Fig. 2*G*) assays, and observed similar N-104 resistance. Thus inner membrane proteins FeoB and PotH and eight other proteins contribute to N-104 bactericidal activity. However, no mutual interaction of the mediators is apparent (BioGRID [v. 4.4] and/or STRING [v. 12]) (Table S2). With all mediators thought to be associated with the inner membrane or cytosol, how is N-104 involved? Does N-104 act directly by gaining access to the periplasm? Or does it act indirectly? We first explored access and then the interaction mechanism and subsequent consequences.

**Table 1:** Direct or indirect mediators of N-104 as per an N-104 forward genetic resistance screen of the *E. coli* Keio collection of 3,884 single gene knockouts

Protein mediator	Function	Ref.
FeoB	inner membrane Fe^2+^ transporter	(4)
PotH	inner membrane polyamine transporter subunit	(31)
RluE	GntR family member YmfC (regulatory)	(38)
YbaE	periplasmic SgrR family member (regulatory)	(36)
YbdM	cytosolic ParB-like nuclease domain containing (regulatory)	(37)
YeeI	zinc metallopeptidase	(34)
YhfZ	putative transcription factor (regulatory)	(39)
YjhQ	growth-promoting antitoxin	(33)
YobD	conserved inner membrane protein	(35)
WcaD	inner membrane colanic acid polymerase	(32)

Forward genetic resistance screens of the Keio *E. coli* 3884 single gene knockout collection in 96 well plate suspension cultures (10^6^ cfu/ml). Mutants with a slope ratio ≥ 0.75 (metabolic slope 5 h after/slope 5 h before 100 μM N-104 treatment) were considered N-104 resistant. Proteins absent in resistant mutants are presumed to be N-104 mediators.

### N-104 outer membrane translocation is facilitated by transporter YaiW

**Figure 3:**
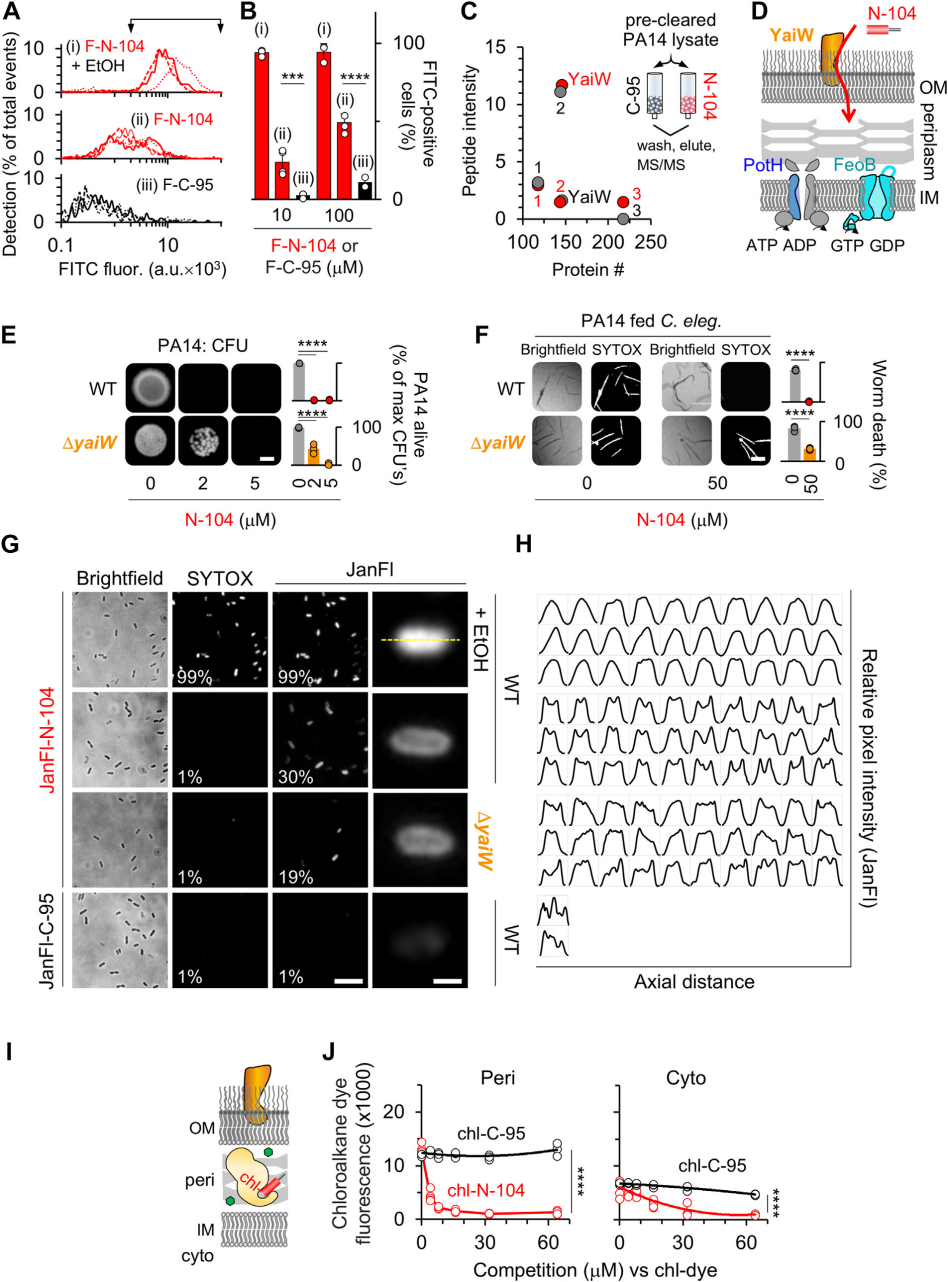
**Outer membrane transporter YaiW helps mediate N-104 translocation into the periplasm.***A*, imaging flow cytometry in which overnight cultures of PA14 (10^6^ cfu/ml) were incubated for 1 h at 35 °C (temperature of eye surface) with (ii) FITC-N-104 or (iii) FITC-C-95, each at 100 μM or with (i) 100 μM FITC-N-104 together with membrane permeabilizing 70% ethanol. Cells were washed, treated with trypsin EDTA to remove surface attached peptide, and then fixed for imaging flow cytometry with analysis by IDEAS (v. 6.2). Arrows indicate gating for *B*. *B*, gated area from *A* together with assays using 100 μM FITC-N-104 and FITC-C-95 ([mean with S.D.], n = 3, ∗∗∗∗*p* < 0.0001; ∗∗∗*p* < 0.001 [two-way ANOVA with Šidák's multiple comparisons test]). *C*, affinity pulldown/tandem mass spectrometric (AP/MS) analysis of PA14 outer membrane proteins with affinity for N-104. PA14 pellets from overnight cultures were lysed in buffered 200 mM *n*-octyl-β-D-glucopyranoside with protease inhibitors, passed through an agarose pre-column (pre-cleared) onto SulfoLinkCys(N)-N-104 or -C-95 columns at 4 °C. Columns were thoroughly washed and then subjected to step gradient elution in the same buffer containing 50, 75, 100, 125, 300, 500, and 1000 mM KCl. Shown are outer membrane proteins from the 500 mM eluant off each column (*red*: N-104; grey: C-95): YaiW; 1, OmpH; 2, OprF; 3, AprF. N-104 binds YaiW, whereas C-95 binds OprF. *D*, schematic diagram of PA14 outer and inner membrane separated by the periplasm and hypothetical YaiW-mediated translocation of N-104 through the outer membrane to reach the periplasmic surface of inner membrane FeoB and PotH. *E*, CFU assay of overnight cultures of wild type and PA14 Δ*yaiW* (10^6^ cfu/ml) treated with 0, 2, or 5 μM N-104. The scale bar is 2 mm. *Right*, quantitation ([mean with S.D.], n = 3, ∗∗∗∗*p* < 0.0001 [two-way ANOVA with Tukey's multiple comparisons test]). *F*, *C. elegans* survival involving 0 or 50 μM N-104 5 h treated wild type and PA14 Δ*yaiW* (10^7^ cfu/ml) that were fed to 50 worms in 96 well plates for 3 days. The scale bar is 200 μm. *Right*, quantitation ([mean with S.D.], n = 3, ∗∗∗∗*p* < 0.0001 [two-way ANOVA with Šidák's multiple comparisons test]). *G*, Brightfield imaging of overnight PA14 (WT) and PA14 Δ*yaiW* cells (∼3.75 × 10^9^ cfu) after incubation with Janelia Fluor549-N-104 or -C-95 for 1 h at 35 °C and simultaneously with 5 μM SYTOX Green. The *dashed yellow line* is the path of an axial tracing analyzed in *H*. Scale bars are 10 (*left*) and 1 (*right*) μm. *H*, single cell axial pixel intensity tracings of Janelia Fluor549-N-104 or -C-95 treated cells. *I*, Bacterial ChloroAlkane Penetration Assay schematic showing YaiW (*orange*) in the outer membrane together in the periplasm with chloroalkane tagged-N-104 (chl-N-104; *red cylinder*) bound to a HaloTag periplasmic protein (*light orange*). Also shown is rhodamine chloroalkane (*green circles*) that competes with chl-N-104 for the HaloTag periplasmic protein. *J*, Bacterial ChloroAlkane Penetration Assay in which *E. coli* (4 × 10^9^ cfu/ml) expressing the HaloTag periplasmic protein (HaloTag Peri; *left*) or HaloTag cytoplasmic protein (HaloTag Cyto; *right*) were incubated for 30 min at 37 °C with 0 to 64 μM of chl-N-104 or -C-95 followed by rhodamine chloroalkane, then washed, fixed and analyzed by flow cytometry. ([mean with S.D.], n = 3, ∗∗∗∗*p* < 0.0001 [two-way ANOVA with Šidák’s multiple comparisons test]).

To determine whether N-104 gains access to the periplasm, we employed imaging flow cytometry. Imaging flow cytometry fuses high throughput features of flow cytometry with single-cell fluorescent microscopy. We synthesized N-104 with FITC on its N-terminus and did the same for negative control C-95, knowing that C{PEG2]-Cy3 tagging of the C-terminus of lacritin N-94/C-6 is inactivating but not when added to the N-terminus (10). Like N-80/C-25, the C-95 synthetic peptide derives from an inactive region of lacritin (Fig. 1*A*). After incubating PA14 cells with each for 1 h, then washing and treating with trypsin to remove surface-attached peptide, cells were fixed and subjected to imaging (Fig. 3, *A* and *B* and Table S3). By first treating cells with FITC-N-104 in the presence of membrane permeabilizing ethanol we established how to gate for intracellular fluorescence (Fig. 3*A*, downward arrows). FITC-N-104 and FITC-C-95 (Fig. 3*A*) scans were then similarly gated for quantitation (Fig. 3*B*). In the absence of ethanol, substantial intracellular FITC-N-104, but not FITC-C-95, fluorescence was apparent at both 100 (Fig. 3, *A* and *B*) or 10 (Fig. 3*B*) μM concentrations. Thus FITC-N-104 crossed the outer membrane. Since N-104 appeared to be incapable of membrane disruption (Fig. 1) and FITC was not membrane permeable, we next explored the possible role of an outer membrane transporter – keeping in mind that surface aggregation or limited membrane translocation might contribute.

No evidence exists for a PA14 transporter of antimicrobial peptides. PA14 genome screening had identified ABC transporters of peptide-opine-nickel, di- and tripeptides and peptidyl nucleoside antibiotics; however, these failed to transport the four tested antimicrobial peptides (41). To explore the existence of such a transporter, we initially considered outer membrane protein biotinylation for attempted N-104 affinity purification followed by tandem mass spectrometry, as was successful in our discovery of the lacritin coreceptor from mammalian cell *n*-octyl-β-D-glucopyranoside lysates (42). However, this approach is complicated by biotin permeation through bacterial outer membrane porin channels (43). There is also the possibility that biotinylation could alter binding properties (44). Precleared whole cell PA14 *n*-octyl-β-D-glucopyranoside lysates were incubated overnight (4 °C) with bead immobilized N-104 or C-95 in 50 mM Tris, 1 mM MgCl_2_ and 200 mM *n*-octyl-β-D-glucopyranoside (pH 7.2), with protease inhibitor cocktails (Fig. 3*C*). *N*-octyl-β-D-glucopyranoside was previously found to optimally refold bacterial outer membrane OmpG (45). Following washes in 50 mM Tris, 50 mM *n*-octyl-β-D-glucopyranoside (pH 7.2), and 20 mM KCl, and progressive step gradient elution through 50, 75, 100, 125, 150, 300, and 500 mM KCl for stringency in the same buffer, the 500 mM KCl eluate was subjected to tandem mass spectrometry. Figure 3*C* compares the N-104 and C-95 500 mM KCl eluates respectively in red and grey. Four outer membrane proteins were identified in both: OmpH (1), OprF (2), AprF (3), and YaiW. Only the lipoprotein YaiW displayed a selective affinity for N-104 (Fig. 3, *C* and *D*). YaiW together with inner membrane SbmA facilitates the nonlytic entry of arginine and proline-rich bovine cathelicidin antimicrobial peptide (Bac7(1–35)) into *E. coli* (46). Indeed, in our N-104 resistance screen, both ΔYaiW (0.70) and ΔSbmA (0.69) *E. coli* were just below the arbitrary resistance cutoff of 0.75. SbmA is absent from PA14 and BLAST failed to detect any homologs; however, the PA14 transposon mutant lacking YaiW was partially resistant both in CFU (Figs. 3*E* and S3*B*) and *C. elegans* survival (Fig. 3*F*) assays.

In enteric bacteria, SbmA contributes to the periplasm-to-cytoplasm translocation of Bac7(1–35) and other antimicrobial peptides (46). Lacking SbmA, N-104 might accumulate in the periplasm. To explore this possibility, we chose brighter and more stable Janelia Fluor 549 to label the N-termini of N-104 and C-95. PA14 was treated with each for 1 h, washed, transferred to agarose pads, and then examined by wide-field fluorescence. We observed a peripheral internal ring of Janelia Fluor-N-104 staining (Fig. 3*G*), as documented by pixel intensity measurements along the axial distance of individual cells (Fig. 3*H*). This pattern is similar to that observed for GFP targeted to the periplasm of *E. coli* by a TorA signal sequence (47). Internal to the ring (presumably the cytoplasm) fluorescence was apparent, but less so. In the absence of YaiW, the frequency of stained PA14 cells was substantially less. Janelia Fluor-C-95 cells lacked staining. After ethanol permeabilization (48), Janelia Fluor-N-104 was almost entirely cytoplasmic. Since the Janelia Fluor 549 label is membrane permeable, we also monitored PA14 for SYTOX Green uptake. None was detected except in the presence of ethanol (Fig. 3*G*), further validating the lack of N-104-dependent membrane disruption.

To scrutinize the apparent periplasmic accumulation of N-104 using a different approach we employed the newly developed Bacterial ChloroAlkane Penetration Assay (BaCAPA) which is currently available only in *E. coli* (49). N-104 and C-95 were N-terminally tagged with chloroalkane. Chloroalkane covalently interacts with a haloalkane dehydrogenase, referred to as HaloTag (50). *E. coli* overexpressing a HaloTagged periplasmic marker (Fig. 3*I*) or in other cells, a HaloTagged cytoplasmic marker were incubated for 30 min with chloroalkane tagged N-104 or C-95, washed, and then treated for 30 min with rhodamine chloroalkane. Rhodamine chloroalkane competes with chloroalkane tagged N-104 or C-95 for binding to the HaloTagged periplasmic or cytoplasmic marker. Chloroalkane-N-104, but not chloroalkane-C-95, effectively inhibited rhodamine chloroalkane targeting of the HaloTag periplasmic marker (Fig. 3*J*) thereby confirming that N-104 is periplasmic. The same was true of the HaloTag cytoplasmic marker, although less substantial (Fig. 3*J*), in keeping with some cytoplasmic Janelia Fluor-N-104 (Fig. 3, *G* and *H*). N-104 thus primarily targets the periplasm with some also in the cytoplasm. Passage across the outer membrane involves YaiW, possibly assisted by N-104 surface aggregation or partial membrane translocation.

### N-104 binds FeoB and PotH ion channels

**Figure 4:**
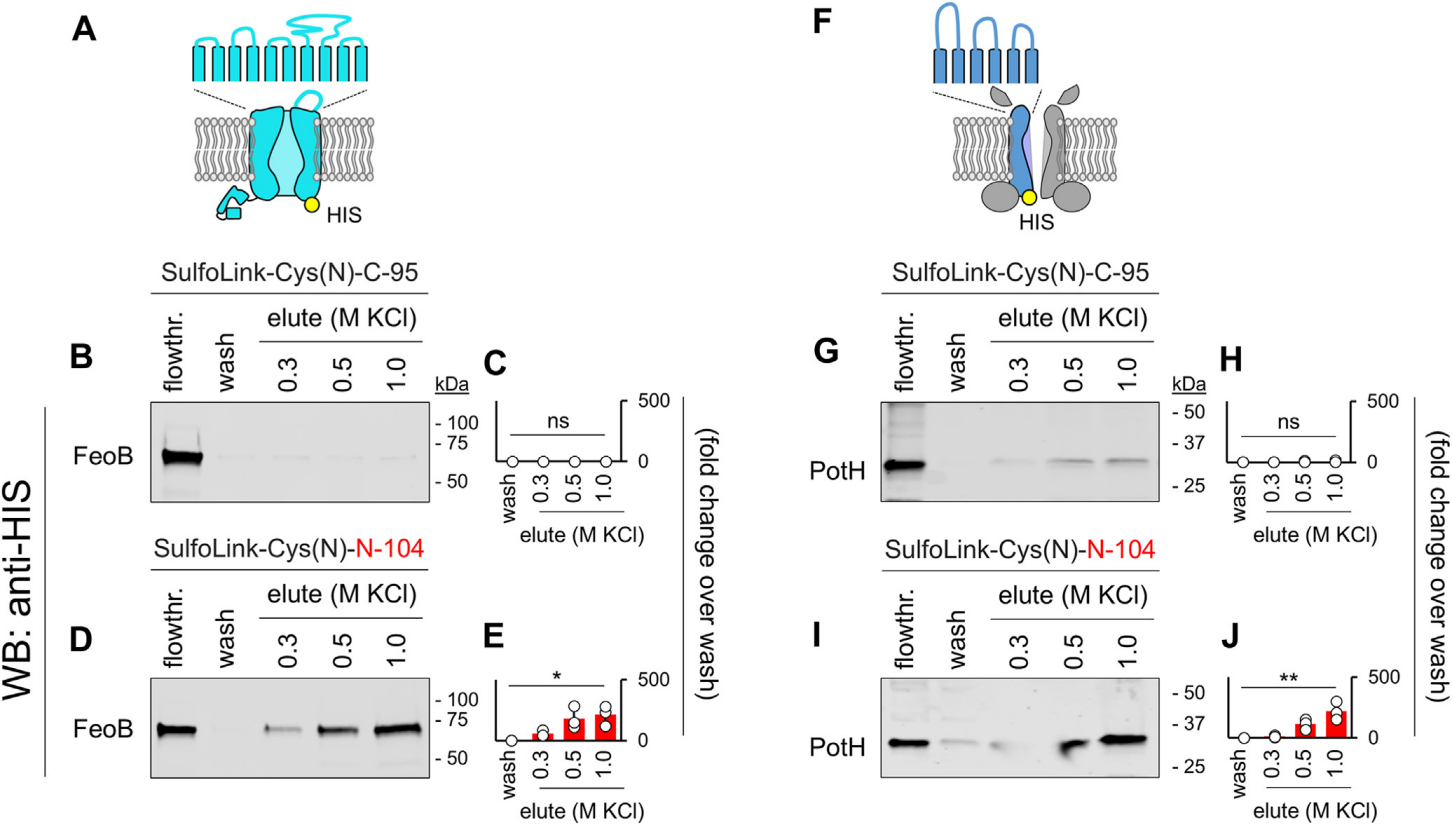
**Inner membrane Fe^2+^ and polyamine transporters FeoB and PotH respectively bind N-104.***A*, schematic of His-tagged FeoB within the inner membrane. Only large outer loop four is depicted and transmembrane domains are not separated. *B*, His-tagged FeoB binding assay with negative control SulfoLinkCys(N)-C-95 columns. Overnight cultures of *E. coli* Lemo21 (DE3) cells overexpressing His-tagged FeoB were lysed in buffered 2% dodecyl-β-D-maltopyranoside with protease inhibitors, captured on nickel columns, eluted, subjected to a buffer change and then passed onto a SulfoLinkCys(N)-C-95 column at 4 °C. Shown is the flowthrough (flowthr), last wash fraction and 0.3 to 1 M KCl elution fractions as detected by anti-His Western blotting. *C*, LI-COR quantitation of (*B*). ([mean with S.D.], n = 3, ns, not significant [Friedman ANOVA]. *D*, the same procedure was performed as in *B*, but with SulfoLinkCys(N)-N-104 columns. *E*, LI-COR quantitation of (*D*) ([mean with S.D.], n = 3, ∗*p* < 0.05; [Friedman ANOVA]). *F*, Schematic of His-tagged PotH subunit as part of the inner membrane PotFGHI transporter. *G*, His-tagged PotH binding assay with negative control SulfoLinkCys(N)-C-95 columns. Overnight cultures of PA14 overexpressing His-tagged PotH were lysed in buffered 2% dodecyl-β-D-maltopyranoside with protease inhibitors, captured on nickel columns, eluted, subjected to a buffer change, and then passed onto a SulfoLinkCys(N)-C-95 column at 4 °C. Shown is the flowthrough (flowthr), last wash fraction, and 0.3 to 1 M KCl elution fractions as detected by anti-His Western blotting. *H*, LI-COR quantitation of *G* ([mean with S.D.], n = 3, ns, not significant [Friedman ANOVA]). *I*, the same procedure was performed as in *G*, but with SulfoLinkCys(N)-N-104 columns. *J*, LI-COR quantitation of *I* ([mean with S.D.], n = 3, ∗∗*p* < 0.01, [Friedman ANOVA]).

By entry into the periplasm, N-104 gains access to periplasmic domains of inner membrane proteins. Inner membrane FeoB is predicted to extend five (TMHMM v 2; Alphafold3) or four (DeepTMHMM 1.0.24; lacks loop 2) loops (Fig. S4*A*) into the periplasm anchored respectively by ten or eight transmembrane domains. This forms a Fe^2+^ transport channel. FeoB's GTPase and GDP dissociation inhibitor domains reside on the cytoplasmic side of the inner membrane. PotH together with PotI constitute the transmembrane polyamine channel of the ABC transporter PotFGHI (51). PotH projects three predicted (DeepTMHMM 1.0.24; TMHMM v 2; Alphafold3) periplasmic loops (Fig. S4*B*) associated with six transmembrane domains. The channel primarily transports spermidine in *P. aeruginosa* and putrescine in *E. coli*. We next generated recombinant His-tagged FeoB and PotH for N-104 affinity studies. We chose to solubilize His-tagged FeoB in the non-ionic detergent dodecyl-β-D-maltopyranoside, as per a prior FeoB detergent screen in which dodecyl-β-D-maltopyranoside optimally conserved FeoB's GTPase activity as did ocetaethylene glycol monododecyl ether and NV10 (52). We screened His-tagged PotH in dodecyl-β-D-maltopyranoside, *n*-octyl-β-D-glucopyranoside, and lauryl maltose neopentyl glycol. Dodecyl-β-D-maltopyranoside was superior (Fig. S4*C*). Nickel-Sepharose enriched His-FeoB and His-PotH were mixed overnight at 4 °C with bead immobilized N-104 or negative control C-95 in 10 mM HEPES containing 0.05% dodecyl-β-D-maltopyranoside, 10% glycerol, 10 mM MgSO_4_ and 0.15 M NaCl (pH 7.4). After collecting the flow through, columns were thoroughly washed and then subjected to a step gradient of 0.3, 0.5, and 1.0 M KCl in the same buffer. C-95 KCl eluates lacked His-FeoB (Fig. 4, *B* and *C*) or His-PotH (Fig. 4, *G* and *H*). This was not the case for N-104 from which His-FeoB (Fig. 4, *D* and *E*) or His-PotH (Fig. 4, *I* and *J*) were eluted with 0.5 and 1 M but not 0.3 M KCl, suggesting a direct interaction between His-FeoB and N-104 or between His-PotH and N-104.

### By targeting FeoB and PotH, N-104 suppresses Fe^2+^ and polyamine uptake

**Figure 5:**
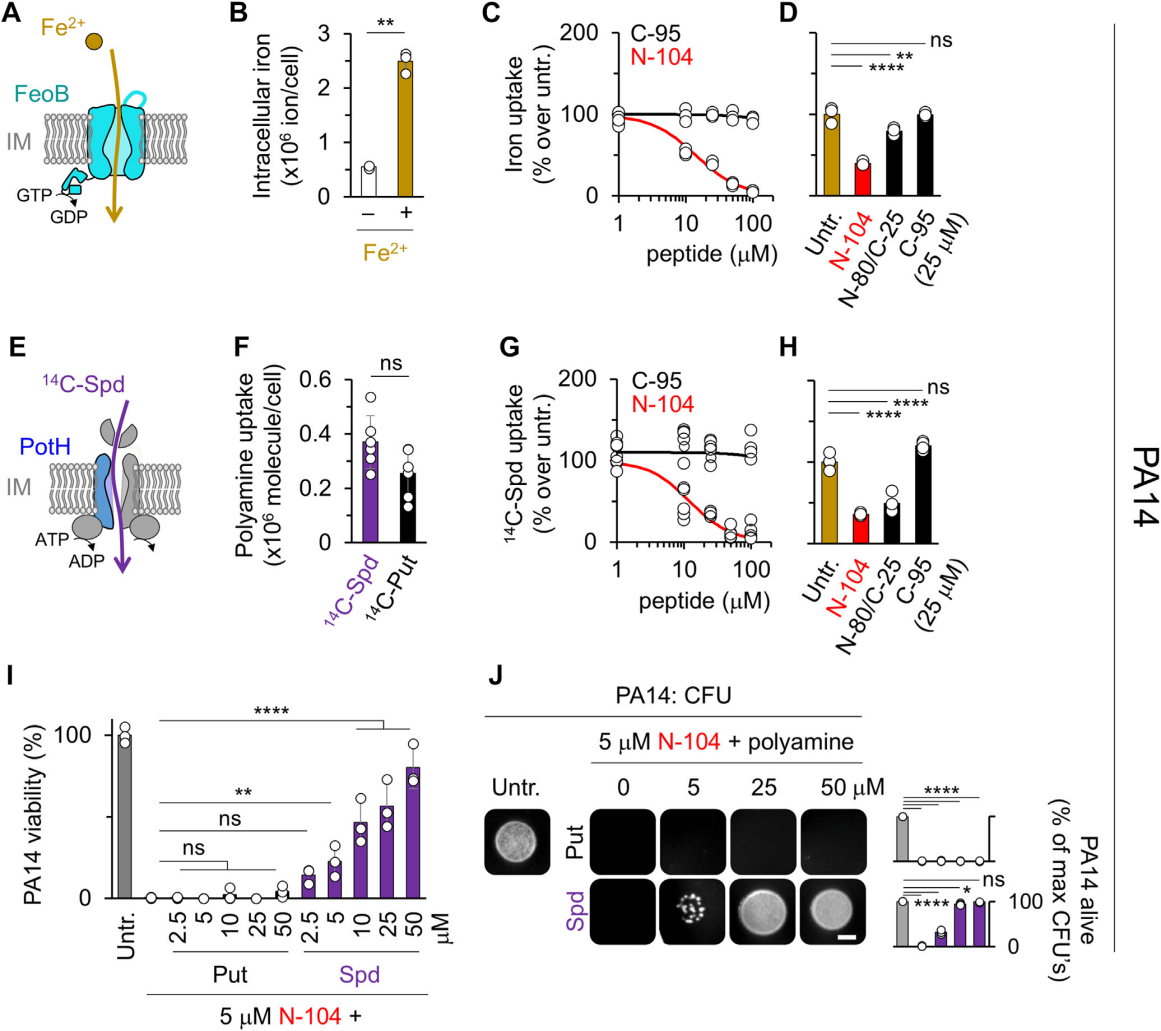
**N-104 suppresses Fe^2+^ and polyamine uptake.***A*, Schematic of Fe^2+^ uptake by FeoB. *B*, Fe^2+^ uptake assay in which overnight cultures of PA14 (5 × 10^9^) were depleted of iron with 1 mM 2,2′-bipyridyl then washed and either left untreated (−) or were incubated with 100 μM of FeSO_4_ (+) for 15 min at 35 °C. For detection of intracellular Fe^2+^, 50 mM NaOH lysates were processed into Fe^2+^ chelator Ferrozine thereby creating a magenta color in supernatants that was detectable at OD_565_. The same assay was used in *C* and *D* ([mean with S.D.], n = 3, ∗∗*p* < 0.01 [unpaired *t* test with Welch's correction]). *C*, Fe^2+^ uptake assay of 2,2′-bipyridyl chelated PA14 (5 x 10^9^) in the presence of 1 to 100 μM N-104 or C-95. Iron was detected using the method in *B*. *D*, chelated PA14 Fe^2+^ uptake assay as per *B* comparing the effect of 25 μM N-104, N-80/C-25 or C-95 *versus* untreated cells ([mean with S.D.], n = 3, ∗∗∗∗*p* < 0.0001, ∗∗*p* < 0.01; ns, not significant [two-way ANOVA with Dunnett’s multiple comparisons test]). *E*, schematic of ^14^C-spermidine (^14^C-Spd) uptake by PotFGHI. *F*, ^14^C-spermidine *versus*^14^C-putrescine (^14^C-Put) uptake assay in which overnight cultures of PA14 (0.8 × 10^8^) were incubated with 10 μM [1,4-^14^C]putrescine or [1,4-^14^C]spermidine for 15 min at 35 °C and then subjected to scintillation counting. The same assay was used in *G* and *H* ([mean with S.D.], n = 3; ns, not significant [unpaired *t* test]). *G*, ^14^C-spermidine uptake assay of PA14 (0.8 × 10^8^) in the presence of 1 to 100 μM N-104 or C-95. ^14^C-spermidine was detected by scintillation counting. *H*, PA14 ^14^C-spermidine uptake assay as per *G* comparing the effect of 25 μM N-104, N-80/C-25, or C-95 *versus* untreated cells ([mean with S.D.], n = 6; ∗∗∗∗*p* < 0.0001, ns, not significant [unpaired *t* test]). *I*, PA14 Alamar *Blue* viability assay in which overnight cultures of PA14 (10^6^ cfu/ml) either untreated or treated in suspension with 5 μM N-104 in the presence of 0 to 50 μM putrescine or spermidine overnight ([mean with S.D.], n = 3; ∗∗∗∗*p* < 0.0001, ∗∗*p* < 0.01, ns, not significant [two-way ANOVA with Dunnett’s multiple comparisons test]). *J*, CFU assay with overnight cultures of PA14 (10^6^ cfu/ml) were either untreated or treated in suspension with 5 μM N-104 in the absence of the presence of 5, 25, and 50 μM putrescine or spermidine for 8 hours and then applied to LB agar for overnight growth. The scale bar is 2 mm. *Right*, quantitation ([mean with S.D.], n = 3, ∗∗∗∗*p* < 0.0001; ∗*p* < 0.05; ns, not significant [two-way ANOVA with Dunnett's multiple comparisons test).

To explore whether N-104 ligation of FeoB and PotH is negatively coupled with iron and polyamine uptake (Fig. 5), we performed iron (53) and both spermidine and putrescine uptake assays. Examination of iron uptake (Fig. 5*A*) was preceded by chelation of PA14 intracellular iron with 2,2′-bipyridyl. Cells were then washed and treated with N-104 or control peptides prior to incubation with ferrous sulfate in the presence of ascorbic acid. Ascorbic acid prevents the oxidation of Fe^2+^ to Fe^3+^. After washing, Fe^2+^ uptake was determined from acidified KMnO_4_ lysed cells through the chelation-dependent colorimetric change in Ferrozine (54). Fe^2+^ binding of Ferrozine creates a magenta color. Uptake was (0.55 ± 0.03) × 10^6^ without and (2.49 ± 0.19) × 10^6^ Fe^2+^ ions per cell with exogenous Fe^2+^ (Fig. 5*B*) as quantitated using an OD_565_ standard curve (Fig. S5*A*). Subsequent treatment with increasing concentrations of N-104 inhibited exogenous Fe^2+^ uptake with an IC50 of 10 μM, whereas C-95 had no effect (Fig. 5*C*). We also tested negative control peptide N-80/C-25 that, like N-104, is cationic. N-80/C-25 was partially inhibitory (Fig. 5*D*) and was slightly inhibitory in CFU assays (Fig. S5*B*).

Polyamine uptake was initially monitored using ^14^C-spermidine and ^14^C-putrescine (Figs. 5, *E* and *F* and S5*C*). After incubation with PA14 for 15 min, cell pellets were thoroughly washed and then assessed for radioactivity. No significant difference was apparent in uptake (Fig. 5*F*). To explore the effect of N-104 and C-95, cells were treated for 45 min with N-104 or C-95 at increasing concentrations prior to a 15 min incubation with ^14^C-spermidine. N-104 inhibited ^14^C-spermidine uptake with an IC50 of 10 μM, whereas C-95 had no effect (Fig. 5*G*), the former in keeping with less sperimidine and putrescine detected in *E. coli* after treatment with recombinant lacritin N-65 (15). Cationic N-80/C-25 (25 μM) was also inhibitory (Fig. 5*H*), although not membrane disruptive (Fig. S5*D*).

In *E. coli* spermidine displays dual roles dependent on concentration. At low micromolar levels, it decreases reactive oxygen species to counter oxidative stress, whereas at millimolar levels, it oxidizes iron to promote the formation of toxic superoxide radicals (55). In keeping with this observation, PA14 cells treated with exogenous spermidine over a 5 to 50 μM concentration range were increasingly protected against 5 μM N-104 killing (Figs. 5, *I* and *J* and S5*E*), whereas at concentrations exceeding 100 μM, spermidine was toxic (Fig. S5, *F* and *G*). Putrescine did not offer the same benefit, nor was toxic at high concentrations (Fig. S5, *F* and *G*). Further, we observed how increasing the concentration of exogenous spermidine up to ∼75 μM chelates thereby increasing the solubility and detectable absorbance of iron (Fig. S5*H*) but not of Mg^2+^, Ca^2+^, or Co^2+^ (Fig. S5*I*). Taken together, N-104 but not C-95, blocks the uptake of Fe^2+^ and spermidine. Cationic negative control N-80/C-25 is also inhibitory, especially for spermidine uptake, without substantially affecting PA14 viability but is not membrane disruptive.

### N-104 synergizes with thrombin antimicrobial peptide GKY20

**Figure 6:**
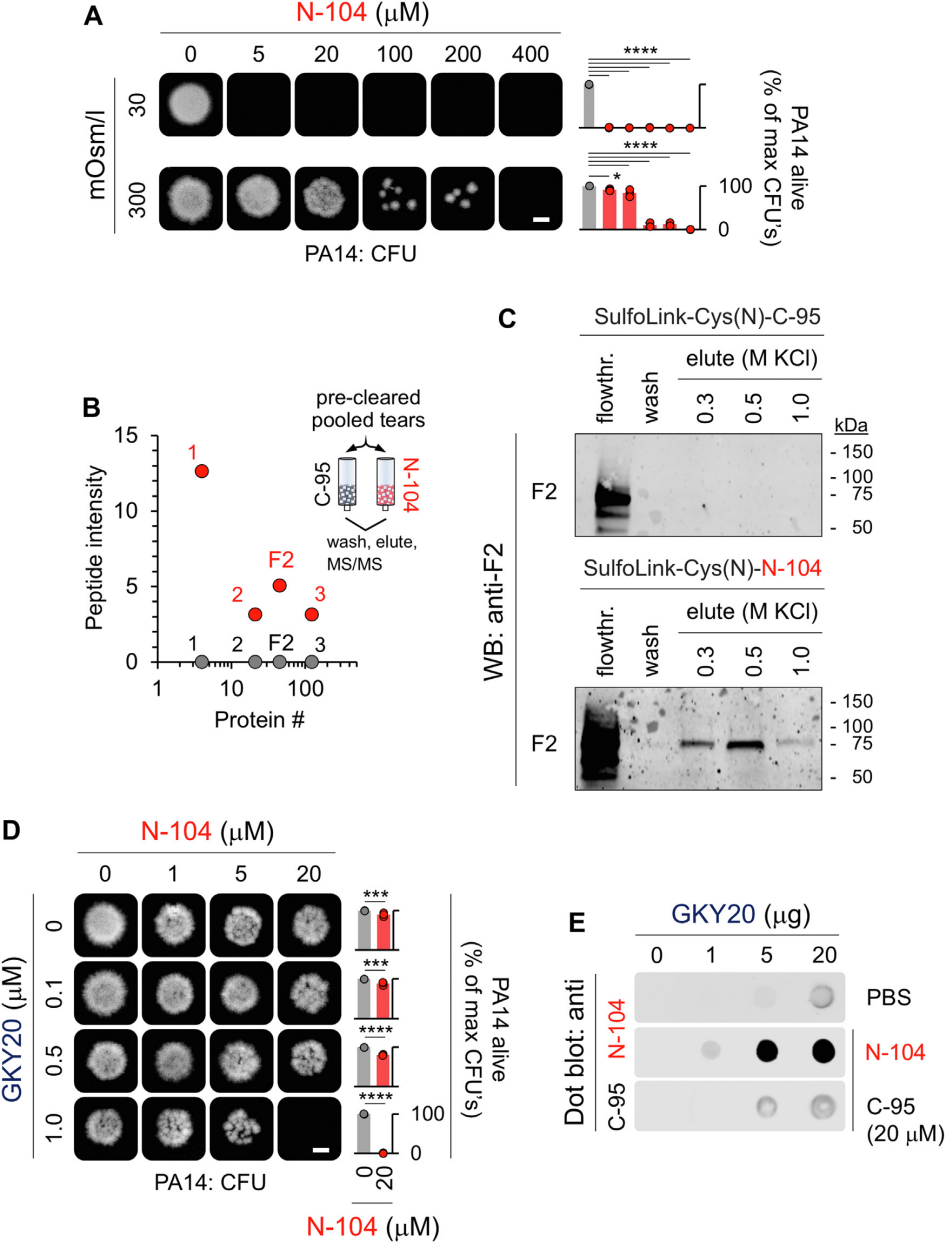
**N-104 protein affinity screen of antimicrobial-rich human tears captures thrombin GKY20 antimicrobial synergy with N-104.***A*, PA14 CFU assay whereby overnight cultures (10^6^ cfu/ml) were incubated in suspension for 8 h with 0 to 400 μM N-104 in 30 or 300 mOsm/l phosphate buffer and then plated onto LB agar. The scale bar is 2 mm. *Right*, quantitation ([mean with S.D.], n = 3, ∗∗∗∗*p* < 0.0001; ∗*p* < 0.05 [two-way ANOVA with Sikak's multiple comparisons test). *B*, affinity pulldown/tandem mass spectrometric (AP/MS) analysis for human tear antimicrobial proteins with affinity for N-104. Pooled human basal tears (170 μl) in 300 mOsm/l phosphate buffer containing protease inhibitors were precleared through an agarose pre-column onto SulfoLinkCys(N)-N-104 or SulfoLinkCys(N)-C-95 columns at 4 °C. Columns were thoroughly washed with 300 mOsm/l phosphate buffer and then subjected to step gradient elution in the same buffer containing 0.3, 0.5, and 1 M KCl. Shown are tear antimicrobial proteins from the 0.5 M eluant off each column (red: N-104; gray: C-95): F2 (thrombin); 1, lacritin (LACRT); 2, gelsolin (GSN); 3, cystatin S (CST4). *C*, immunodetection of F2 (thrombin) in the 0.5 M KCl eluant off SulfoLinkCys(N)-N-104 but not SulfoLinkCys(N)-C-95 columns. Equal volumes of the flow through (flowthr), final wash fraction, and KCl elution fractions from *B* were subjected to 4 to 20% SDS-PAGE without sample boiling for anti-F2 immunodetection using LI-COR. *D*, PA14 CFU checkerboard assay using the same method as in *A* wherein PA14 was incubated with mixtures of 0 to 20 μM N-104 and 0 to 1 μM GKY20 in 300 mOsm/l phosphate buffer and then plated onto LB agar. The scale bar is 2 mm. *Right*, quantitation ([mean with S.D.], n = 3, ∗∗∗∗*p* < 0.0001; ∗∗∗*p* < 0.001 [two-way ANOVA with Sikak's multiple comparisons test). *E*, dot blot binding assay in which 0 to 20 μg of GKY20 was immobilized on nitrocellulose, blocked with 1% fish gelatin and then incubated with 20 μM N-104 or C-95 for 2 h at 35 °C for anti-N-104 or anti-C-95 LI-COR detection.

N-104 plays a central role in the innate defense of the eye as a cleavage-potentiated lacritin fragment (15). Likely it does not act in isolation. Synergism among antimicrobial peptides, some with remarkable functional specificity, is well established (56) and at least twenty-two other putative or demonstrated antimicrobial proteins have been detected in human tears (57). Many are also in plasma, cerebral spinal fluid, and saliva. Further, it is apparent that the efficacy of N-104 diminishes as osmolarity increases (Figs. 6*A* and S6*A*) despite its high affinity for YaiW, FeoB, and PotH (which are fully elutable only at 500 mM KCl); and earlier evidence with the larger N-65 fragment (15). To explore possible synergies, we subjected precleared human tears to bead-immobilized N-104 or C-95. Following extensive washing, 500 mM KCl eluants were subjected to tandem mass spectrometry (Fig. 6*B*). N-104 interacted with ten tear antimicrobial proteins (MassIVE MSV000094085). Of these, we focused on three whose literature data was considered strong. Figure 6*B* compares the N-104 (red) and C-95 (grey) 500 mM KCl eluates of: thrombin (F2; Fig. 6*C*), gelsolin (2), and cystatin S (3), each respectively containing the antimicrobial: GKYGFYTHVFRLKKWIQKVI known as GKY20 (58), QRLFQVKGRR (59) and SSSKEENRIIPGGI (60) peptides. Lacritin was also detected (1). GKY20 overlaps with antimicrobial FYT21 released from thrombin by *P. aeruginosa* elastase (61). Each synthetic peptide was synthesized. We then challenged PA14 with increasing concentrations of both N-104 and one of each of the three peptides in checkerboard CFU assays. Osmolarity was 300 mOsm/l. Only GKY20 was synergistic (Figs. 6*D* and S6*B*), consistent with its carpet-like mechanism of lipid disruption which has been studied in model membranes (62). N-104 binds thrombin (Fig. 6*C*). Might it directly interact with GKY20 in thrombin's C-terminus? To address this question, GKY20 was immobilized on nitrocellulose, blocked, and then overlayed with 20 μM N-104 or negative control C-95 for 2 h. Only N-104 bound to GKY20 (Figs. 6*E* and S6*C*). This assay was repeated with each of the 10 N-104 serine-substituted analogs shown in Figure 1*A*. Single serine substitution did not disrupt the interaction (Fig. S6*D*). N-104 containing six serine substitutions (L108, L109, L114, L115, F112 and W118; LFW:S) could slighly disrupt N-104 interaction (Fig. S6, *E* and *F*), but was not bactericidal (Fig. S6*G*). The interaction may therefore be electrostatic. Thus, tear N-104 shares affinity for and synergizes with tear GKY20 for improved bactericidal efficacy under physiological conditions.

### N-104 - GKY20 efficacy against clinical isolates

**Figure 7:**
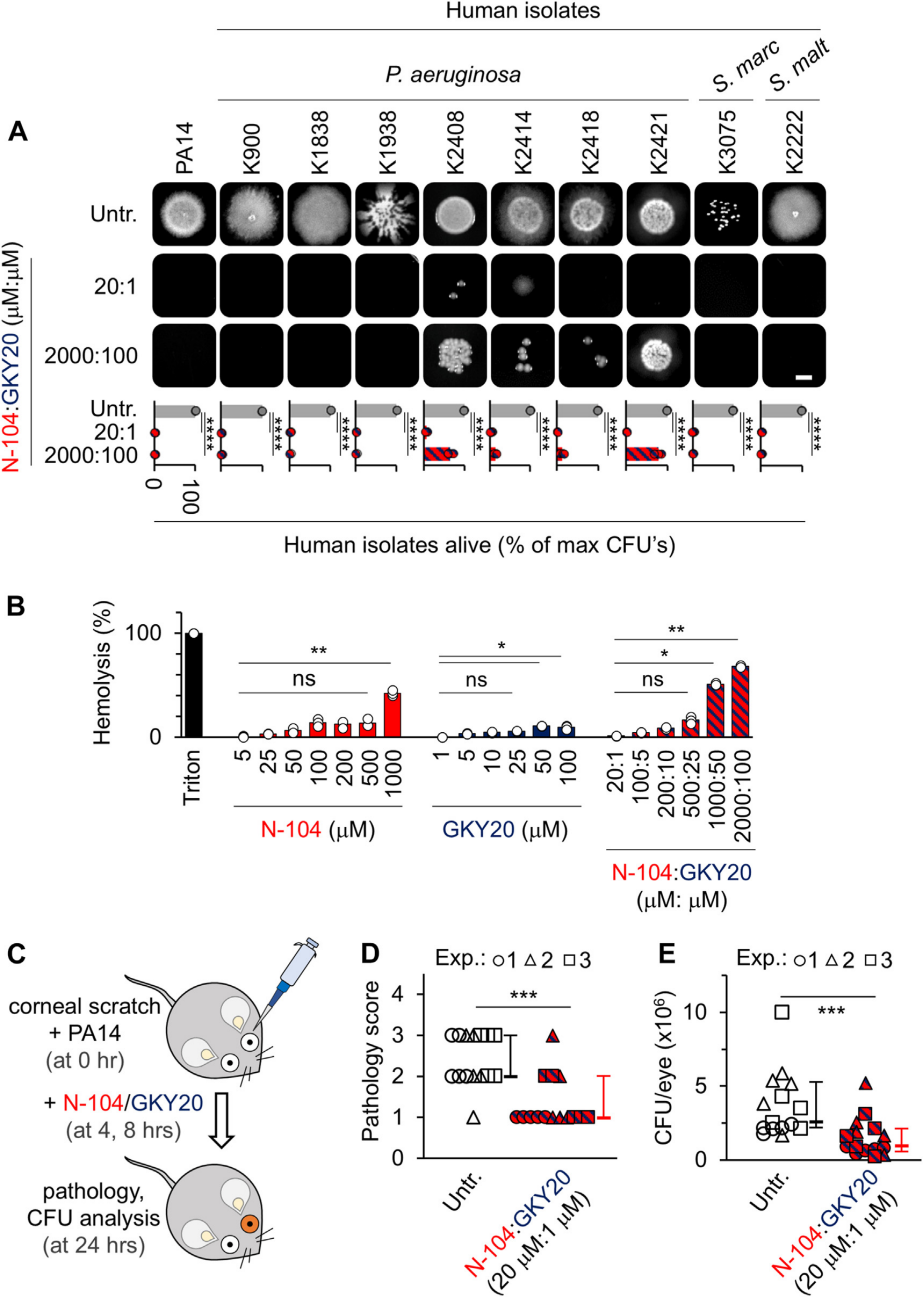
**N-104 - GKY20 synergy kills nine multidrug-resistant human clinical isolates, and PA14 on infected mouse eyes.***A*, colony-forming unit assay whereby overnight cultures of PA14 or of each human clinical isolate were incubated in suspension for 8 h without (Untr.; 10^3^ cfu/ml) or with 20:1 μM:μM (10^3^ cfu/ml) or 2000:100 μM:μM (10^5^ cfu/ml) of N-104:GKY20 in 300 mOsm/l phosphate buffer and then plated onto LB agar. Scale bar is 2 mm. *Below*, quantitation ([mean with S.D.], n = 3, ∗∗∗∗*p* < 0.0001 [two-way ANOVA with Sikak's multiple comparisons test). *B*, sheep red blood cell hemolysis assays. Positive control 5% Triton X-100 or 5 to 1000 μM N-104, 1 to 100 μM GKY20, or 20:1, 100:5, 200:10, 500:25, 1000:50 or 2000:100 μM mixtures of N-104 and GKY20 were incubated with sheep red blood cells in 300 mOsm/l phosphate buffer for 2 h. Released hemoglobin in the supernatant was detected at OD_540_ ([mean with S.D.], n = 3; ∗∗*p* < 0.01, ∗*p* < 0.1, ns, not significant, [Friedman ANOVA with Dunn's multiple comparisons test]). *C*, schematic of murine corneal infection assay whereby anesthetized mice with three 1-mm scratches on one cornea were infected with 5 μl each of 10^6^ cfu/ml PA14 in 1% proteose peptone. Four and 8 h later, 5 μl of 1 μM N-104:20 μM GKY20 in a buffered solution was applied to the infected eyes. At 24 h, pathology and CFU analyses were performed (63). *D*, eyes were assigned a 0 to 4 pathology score: 0, eye macroscopically identical to the uninfected contralateral control eye; 1, faint opacity partially covering the pupil; 2, dense opacity covering the pupil; 3, dense opacity covering the entire anterior segment; and 4, perforation of the cornea and/or phthisis bulbi ([mean with S.D.], n = 3, ∗∗∗*p* < 0.001, d_Cohen_ = 1.569 [Mann-Whitney test and effect size estimate for nonparametric tests from Psychometrica Computation of Effect Sizes]). *E*, for CFU analysis, corneas removed from euthanized mice were homogenized in 1% proteose peptone containing 0.05% Triton X-100 for serial dilution and bacteria plating on 5% sheep blood plates ([mean with S.D.], n = 3, ∗∗∗*p* < 0.001, d_Cohen_ = 1.479 [Mann-Whitney test and effect size estimate for nonparametric tests from Psychometrica Computation of Effect Sizes]).

We obtained seven different clinical isolates of *P. aeruginosa*, as well as one each of *Serratia marcescens* and *Stenotrophomonas maltophilia* from the University of Pittsburgh's Charles T. Campbell Ophthalmic Microbiology Laboratory. Each is resistant to bacitracin, vancomycin, cefazolin, and sulfasoxazole. Additional resistance is to polymyxin B (*S. marcescens*), gentamicin, tobramycin (*S. maltophilia*), and ofloxacin and moxifloxacin (*P. aeruginosa* K2414). Suspensions from overnight cultures were incubated for 8 h with 20 μM N-104:1 μM GKY20 or 2000 μM N-104:100 μM GKY20, respectively with bacterial densities of 10^3^ cfu/ml and 10^5^ cfu/ml in 300 mOsm/l buffer. N-104/GKY20 was effective against both *S. marcescens* and *S. maltophilia* strains and all but two strains of *P. aeruginosa* (Figs. 7*A* and S7, *A* and *B*). Concentrations higher than 20 μM N-104:1 μM GKY20 were required at higher bacterial densities (Fig. S7, *A* and *B*). Hemolytic activity of N-104 and GKY20 (Fig. 7*B*) alone or together up to 500 μM N-104:25 μM GKY20 was low.

We next evaluated the efficacy of 20 μM N-104:1 μM GKY20 in a well-established mouse model of PA14 corneal inflammation (63). Tears rich in antimicrobial peptides prevent infection of normal eyes but less effectively when the cornea is wounded. Scratches were introduced in corneas of anesthetized mice to facilitate seeding of PA14 four and 8 h before topical treatment with 20 μM N-104:1 μM GKY20 (Fig. 7*C*). Twenty-four hours later, we assessed the severity of keratitis and CFUs. Treatment significantly diminished the level of ocular opacity by ∼2 fold (Fig. 7*D*) and CFU infectivity by ∼2.8 fold (Fig. 7*E*), each with a strong effect size estimate (d > 1.4). Thus, N-104 with GKY20 act synergistically to help kill multidrug-resistant *P. aeruginosa*, *S. marcescens*, and *S. maltophilia in vitro* and PA14 in infected mice.

### N-104 alters the PA14 transcriptome

**Figure 8:**
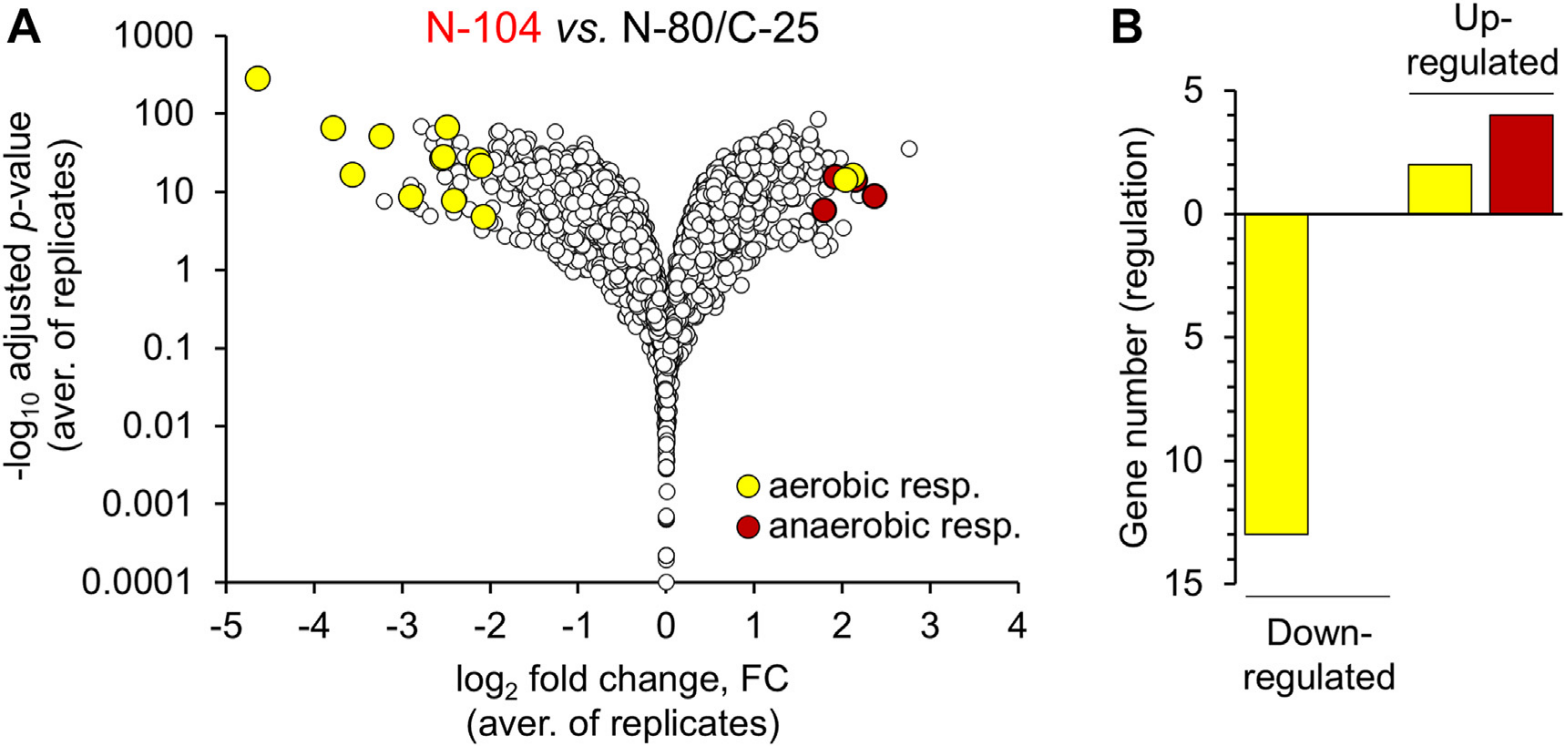
**N-104 suppression of aerobic respiration in favor of FeoB-appropriate anaerobic respiration (although in an aerobic environment) suggested by RNAseq.***A*, Volcano plot of PA14 N-104 differential gene expression *versus* N-80/C-25. Overnight PA14 (5 × 10^8^ cfu/ml) was treated with 20 μM N-104 or N-80/C-25 for 45 min at 35 °C and then processed for RNAseq. *Yellow* and *red* dots respectively represent genes associated with aerobic or anaerobic respiration. *B*, documentation of the number of N-104 up- or down-regulated aerobic (*yellow*) or anaerobic (*red*) genes.

Finally, we surveyed whether N-104 alters the PA14 transcriptome. PA14 cells treated for 45 min with N-104 or N-80/C-25 were subjected to RNAseq (Fig. 8*A*). As mediators of N-104 killing activity, mRNA levels of YaiW, FeoB, and PotH were unaffected. The opposite was true for many others (NCBI GEO DataSets: Accession ID: GSE253123). Downregulated genes included those appropriate for or necessary for aerobic respiration (Fig. 8*B*) including Fe^3+^ transport, such as ExbB (64) and FbpA (65), and electron transport or other parts of the respiratory chain (CoxA, CoxB, EtfA, EtfB, FadH1, PA14_02460, PA14_06640, PA14_25840). Among upregulated genes are those whose proteins are necessary for the anaerobic growth of *P. aeruginosa* in cystic fibrosis (66), including respiratory nitrate reductases (NarG, NarH, NarI, NarJ); and MoeA1 and MoaB1 involved in molybdopterin cofactor biosynthesis (Fig. 8*B*). To determine whether any of these as *E. coli* orthologs may interact with YaiW, FeoB, and/or PotH, we explored BioGRID (Table S2). The only predicted protein interaction was that of FeoB with ExbD (which binds ExbB). Thus with FeoB favoring an anaerobic environment, N-104 promotes the expression of genes regulating anaerobic respiration while largely suppressing those involved in aerobic respiration – a strategy counterproductive under aerobic conditions.

## Discussion

Using a synthetic peptide as a proxy for the cationic, bactericidal N-104 cleavage fragment of plasma, cerebral spinal fluid, tear, and salivary glycoprotein lacritin, we report a novel, endogenous, and non-lytic process to kill *E. coli* and drug-resistant *P. aeruginosa, S. marcescens* and *S. maltophilia* with polypharmacology. N-104 gains periplasmic access *via* outer membrane lipoprotein YaiW to ligate inner membrane ferrous iron transporter FeoB (a well-known virulence factor) and the PotH subunit (possibly *via* outer loop 2 (Fig. S4)) of the PotFGHI polyamine transporter to respectively suppress both ferrous iron and polyamine uptake. Although not yet validated by protein binding and mutational studies, we further report eight other direct or indirect putative targets including inner membrane colanic acid polymerase WcaD involved in biofilm formation, the growth-promoting antitoxin YjhQ of the YjhX-YjhQ toxin-antitoxin system, outer membrane-periplasmic solute-binding protein YbaE, zinc metallopeptidase MtfA (YeeI), inner membrane protein YobD, outer membrane-periplasmic solute-binding protein YbaE, cytosolic ParB-like nuclease domain-containing YbdM, GntR transcription factor family member, pseudouridine synthase RluE and putative transcription factor YhfZ.

Such N-104 multi-target polypharmacology of apparently unrelated pathways was unexpected and may explain the inability of PA14 to develop resistance against N-104 -thereby addressing the 16-year goal of 'realizing the principle of polypharmacology in a single molecular entity' (67). Identification of druggable antibiotics with multiple unrelated target polypharmacology remains a strong need (68). Several have been discovered, but none share N-104's mode of action. For example, Irresistin-16 targets FolA to inhibit folate metabolism and is membrane-disruptive (69). FDA-approved Kibdelomycin targets the type IIA topoisomerases GyrB and ParE essential for DNA transcription and replication (70). Teixobactin binds the respective peptidoglycan and teichoic acid precursors lipid II and III to inhibit cell wall synthesis (71). Cationic β-sheet antimicrobial peptide thanatin is membrane disruptive and also inhibits a common metallo-β-lactamase (72). Others are rationally designed, such as the small molecule dual inhibitor six inspired by an antagonist of the transcriptional regulator PqsR and an inhibitor of PqsD enzyme necessary for the development of the PA14 quinolone signal quorum-sensing system (73), or involve the synthetic linkage of two or more small molecule antibiotics or cationic antimicrobial peptides as hybrids (74). Not apparently common is a noncovalent synergistic protein complex of antimicrobial peptides derived from the same host fluid source, such as with N-104 and tear thrombin peptide GKY20.

N-104's polypharmacology likely centers on its amphipathic α-helix, a common interface for protein-protein and protein-lipid interactions among single-target and multitarget proteins. This is the case for the parent protein lacritin in mammalian cells. The same amphipathic α-helix mediates lacritin's epithelial (12, 75, 76), neuronal (9) regenerative (12, 17), autophagic (11), and secretory (13, 17, 77, 78) activities. Two lacritin synthetic peptides that overlap with N-104 also act as surfactants in the human tear film (10). If N-104's amphipathic α-helix is indeed key, the attenuated bactericidal activity of N-104 with L108S, L109S or F112S substitutions on the polar face of the α-helix could be a consequence of diminished interaction with fatty acid-modified YaiW and with hydrophobic patches on predicted outer loops four and two of the inner membrane transporters FeoB and PotH. The interaction of GKY20 with N-104 containing six serine substitutions at L108, L109, L114, L115, F112, and W118 was only slightly disrupted pointing to a different approach. Like N-104, GKY20 also forms an amphipathic α-helix. Antimicrobial peptides with amphipathic α-helices can self assemble such as the cathelicidin LL-37 (79) or can synergize as heterodimers such as between *X. laevis* magainin 2 and PGLa (80).

YbdM, GntR, RluE, and/or YhfZ might contribute to N-104's alteration of the PA14 transcriptome. Coupled with the transcriptional downregulation of ten other electron transport or respiratory chain or fatty acid oxidation elements by N-104, the apparent suppression of aerobic respiration and inhibition of polyamine transporter PotH is profound. By a different mechanism, cytochrome oxidase-bd of the inner membrane electron transport is thought to be targeted by antimicrobial cationic peptides LL-37 (81) and gramcidin S (82) leading to the generation of toxic superoxide under aerobic conditions. In the anoxic Fe^2+^-rich environment of the Earth’s Paleoarchean period, polyamines are thought to have nourished LUCA—the hypothetical last universal common ancestor (3) from which bacteria, archaea, and eukaryotes evolved. N-104 thus in effect reverses evolutionary time to an anaerobic Fe^2+^- and polyamine-dependent era, thereby suppressing viability in both aerobic and presumably anaerobic environments.

## Experimental procedures

### Cells

The specifications and sources of cells purchased commercially are detailed in the Supporting Information Reagents Table. Seven different clinical isolates of *P. aeruginosa*, as well as one each of *S. marcescens* and *S. maltophilia* were provided by Robert Shanks of the University of Pittsburgh's Charles T. Campbell Ophthalmic Microbiology Laboratory. *P. aeruginosa* PA14 and PA14 *feoB*, *potH (spuG),* and *yaiW* transposon mutants were provided by Mihaela Gadjeva from Harvard University's PA14 Transposon Insertion Mutant Library (40). PA14 is a well-characterized hypervirulent, pathogenic, and multidrug-resistant strain that is commonly employed as a model for *P. aeruginosa* pathogenesis of the eye and in cystic fibrosis. PA14's virulence and pathogenesis are advantageous *versus* the PAO1 strain for these studies. *E. coli* and *P. aeruginosa* were expanded in LB broth. *C. elegans* L1 stage worms were cultivated at room temperature on Nematode Growth Medium (NGM) plates supplemented with *E. coli* OP50 as a food source until they matured into sterile young adults.

### Synthetic peptide design

Synthetic peptides (Supporting Information Reagents Table; all by GenScript (Piscataway NJ)) were each synthesized as trifluoracetic acid (TFA) salts (≥95% pure; HPLC) with N-terminal acetylation (N-104, N-80/C-25) and C-terminal amidation (N-80/C-25, C-95). In *Staphylococcus aureus*, several TFA peptides were superior to the same peptides as acetate or chloride salts, with less hemolysis (83). For imaging flow cytometry studies of N-104 entry into PA14 cells, we, respectively, synthesized FITC-N-104 and FITC-C-95 with N-terminal FITC. Similarly, for higher resolution wide-field fluorescence of the same process, we synthesized Cys(N)-N-104 and Cys(N)-C-95 with an N-terminal added cysteine, to which was conjugated to Janelia Fluor 549 maleimide. Conjugation was initiated in 50 mM Tris (pH 8.5) and 5 mM EDTA at a 10:1 fluorophore to peptide molar ratio for 3 h at room temperature in the dark. Conjugated peptides were separated from free fluorophore by Sephadex G-10 size exclusion chromatography followed by MALDI-TOF analysis of the conjugate fraction. Validation of these experiments utilized the Bacterial ChloroAlkane Penetration Assay for which chloroalkane tagged N-104 and C-95 were synthesized as previously described (49).

To screen for proteins in PA14 lysates and later human tear proteins with affinity for N-104, Cys(N)-N-104 (or negative control Cys(N)-C-95) was immobilized on SulfoLink Coupling resin. Bactericidal peptides from identified tear N-104 binding proteins (thrombin (F2), gelsolin (GSN), and cystatin S peptide (CST4)) were synthesized (see Supporting Information Reagents Table).

Guided by TMHMM v 2 and DeepTMHMM 1.0.24, we synthesized peptides corresponding to PotH’s predicted outer loops 2 (WMGILKNNGVLNNFLLWLGVIDQPLTILHTN) and outer loop 3 (ELLGGPDSIMIGRVLWQEFFNNRDW). Fifty-three amino acid PotH's OL1 was not synthesized with predicted poor solubility. We also synthesized peptides corresponding to FeoB's predicted outer loop 1 (INIGGALQP), outer loop 2 (LEDSGYMARAAFVMDRLMQ), outer loop 3 (GAFFGQGGA), and outer loop 5 (ATFAA). Synthesis of a 131 amino acid FeoB's outer loop four peptide was not attempted. FeoB's outer loop one and three peptides were not water, DMSO, methanol, isopropanol or acetonitrile soluble. Solubility of FeoB’s outer loop one peptide in formic or acetic acid, and outer loop three in ammonia water and acetic acid was identified; however, N-104 - FeoB ligation was not possible in each. We also synthesized human syndecan-1 synthetic peptide ‘pep19 to 30′ peptide, because the lacritin targeting ‘GAGAL' domain in human syndecan-1 synthetic peptide ‘pep19 to 30′ is similar to respective hydrophobic FeoB’s outer loop one and three sequences ‘GGAL' and ‘GA(LA)', with ‘LA' membrane-associated. For these, all amino and carboxy termini were acetylated and amidated, respectively (Supporting Information Reagents Table). All synthesized peptides were aliquoted and stored lyophilized at −80 °C in a dry environment.

### Surface plasmon resonance (SPR)

A lipid bilayer supported on a lipophilic SPR sensor chip L1 (BR100558; GE Healthcare Life Sciences) was prepared using a 59:21:20 mixture of 1-palmitoyl-2-oleoyl-sn-glycero-3-phosphocholine:1-palmitoyl-2-oleoyl-sn-glycero-3-phospho-(1′-rac-glycerol):1-palmitoyl-2-oleoyl-sn-glycero-3-phosphoethanolamine (PC:PG:PE) with some similarity to the outer Gram-negative bacterial membrane although lacking lipopolysaccharide (20). Another lipid bilayer was formed from PC:PG (75:25) as a model for Gram-positive bacteria (21). The lipid bilayers were formed in 8.3 mM phosphate buffer and 10 mM NaCl (pH 7.2). N-104 or N-104 analogs were then introduced to a Biocore 3000 (Cytiva, Marlborough MA) and their time-resolved SPR responses were recorded at a flow rate of 30 μl/min for 100 s, followed by a buffer wash at 30 μl/min for 500 s. The use of a high flow rate minimized mass transfer limitations and resulted in good agreement among the four replicates of each experiment. The experiment was terminated by the addition of 20 mM 3-[(Cholamidopropyl) dimethylammonio]-1-propanesulfonate (CHAPS) to remove lipids and peptides from the sensor chip for reuse. CHAPS was introduced at a flow rate of 5 μl/min for 1 min.

### Colony-forming unit (CFU) assay

100 microliter aliquots of wild-type *E. coli* or *E. coli* gene deletion strain, or wild-type or transposon mutant PA14 overnight cultures were suspended in 1 mM Na_2_HPO_4_, 0.18 mM KH_2_PO_4_, 0.27 mM KCl, 13.7 mM NaCl (pH 7.2) or in 10 mM Na_2_HPO_4_, 1.8 mM KH_2_PO_4_, 2.7 mM KCl, 137 mM NaCl (pH 7.2), at a final density of 10^6^ cfu/ml. Each was treated with N-104, N-80/C-25, C-95, N-104 analogs, N-104 together with thrombin peptide GKY20, or left untreated for ∼8 h at 35 °C (the temperature at the eye surface), and then inoculated (2 μl) on a Luria-Bertani agar plate with or without relevant antibiotic supplementation. After overnight incubation at 37 °C, plates were analyzed on a ChemiDoc XRS (Bio-Rad).

### PA14 - *C. elegans* survival assay

L1 stage *C. elegans* worms on Nematode Growth Medium (NGM) plates with *E. coli* OP50 as a food source were cultivated at room temperature in a humidified chamber until maturation into sterile young adults (84), carefully washed off with M9 minimal salts and then collected by pelleting (1200*g*, 2 min). Approximately 50 worms per well in sterile black 96-well plates containing 150 μl of 90% M9 minimal salts and 10% LB broth were then incubated aerobically with an equal volume of cultures of PA14 or PA14 transposon mutants of *feoB* (PA14_56680), *potH* (PA14_03950), or *yaiW* (PA14_29270), or alternatively with *E. coli* OP50 as negative control (diluted to 10^7^ cfu/ml in 1 mM Na_2_HPO_4_, 0.18 mM KH_2_PO_4_, 0.27 mM KCl, 13.7 mM NaCl (pH 7.2)) that had been previously treated with N-104 or N-104 analogs (50 μM of each) for 5 h at 35 °C. After 3 days, bacteria and debris were removed leaving 50 μl per well of worms to which 50 μl of 6 μM SYTOX Orange was added in M9 minimal salts. The next day, images were captured using both transmission and epifluorescence (excitation/detection = 535/610 nm) on an AMG EVOS FL AMF-4301 fluorescence microscope (Thermo Fisher Scientific).

### Nuclear magnetic resonance and circular dichroism analyses

Two N-104 samples were dissolved in 10 mM Na_2_HPO_4_, 1.8 mM KH_2_PO_4_, 2.7 mM KCl, 137 mM NaCl (pH 7.2) with or without the addition of 150 mM dodecylphosphocholine-d_38_ (DPC-d_38_), respectively. NMR spectra were collected at 25 °C at an N-104 concentration of 1 mM. For both samples, ^1^H-^1^H TOCSY and ^1^H-^1^H NOESY spectra were collected at various mixing times to obtain the best-resolved spectra for spectral assignment. The chemical shifts showed that N-104 in the buffer without dodecylphosphocholine-d_38_ existed only in a random coil structure. In contrast, in dodecyl phosphocholine-d_38_ N-104 showed characteristic helical chemical shifts so that we were able to walk through the resolved linkages in the spectra to assign sequence-specific chemical shifts. The combination of ^1^H-^1^H TOCSY with a mixing time of 60 ms and ^1^H-^1^H NOESY with a mixing time of 80 ms resulted in the proton chemical shift. Subsequently, ^13^C-^1^H and ^15^N-^1^H HSQC spectra were collected by leveraging the naturally abundant ^13^C and ^15^N isotopes in the peptide samples. The near-complete backbone chemical shifts allowed us to use TALOS+ to predict the existence of a helical structure from residues 1 to 10 in N-104 (Fig. S1*A*). Both random coil index order parameters (RCI S2, in block dots) and α-helicity (in red bars) are direct outputs from TALOS+. For NMR validation, N-104 in 10 mM Na_2_HPO_4_, 1.8 mM KH_2_PO_4_, 2.7 mM KCl, and 137 mM NaCl (pH 7.2), without or with 10 mM dodecyl phosphocholine was assayed by circular dichroism in a J-1500 Circular Dichoism Spectropolarimeter (JASCO, Easton MD) over a 190 to 240 nm range.

### Hemolysis assay

The pellet resulting from 300*g* (5 min) centrifugation of 500 μl of stock sheep red blood cells (55876; MP Biomedicals) was resuspended in an equal volume of 10 mM Na_2_HPO_4_, 1.8 mM KH_2_PO_4_, 2.7 mM KCl, 137 mM NaCl (pH 7.2), of which 50 μl aliquots were treated at 37 °C for 2 h with 0, 5, 25, 50, 100, 200, 500 or 1000 μM of N-104 or 1, 5, 10, 25, 50, or 100 μM of GKY20 or both (N-104:GKY20 [μM each]: 20:1, 100:5, 200:10, 500:25, 1000:50, 2000:100) or as a positive control with 5% Triton X-100 in a final volume of 100 μl. After centrifugation at 300*g* for 5 min, the supernatant absorbance at 540 nm of the supernatant minus the absorbance of the untreated sample was recorded and normalized to that of the Triton X-100 sample.

### Forward genetic screens of the Keio *E. coli* K-12 single gene knockout collection

We took advantage of the Alamar Blue viability assay (85). Briefly, wells of deep 96-well plates (1896–2000, USA Scientific) each containing 0.8 ml of LB broth supplemented with 50 μg/ml of kanamycin (BP906–5, Fisher Scientific) were singly inoculated with 10 μl aliquots of each of the 3884 Keio *E. coli* K-12 single gene knockout strains for 16 h at 37 °C on a rotator (160 rpm). A total of 200 μl of a 1:1000 dilution of each (∼10^6^ cfu/ml) in 1 mM Na_2_HPO_4_, 0.18 mM KH_2_PO_4_, 0.27 mM KCl, 13.7 mM NaCl (pH 7.2) containing 10% Alamar Blue (DAL1100, Invitrogen) in wells of black 96-well plates was monitored hourly for 5 h in a SpectraMax M3 plate reader (Molecular Devices) with 3 s agitation before simultaneously reading from the well bottoms at medium PMT voltage with a 590 nm cutoff filter and six flashes per read for the Alamar Blue fluorescence signal (excitation/detection = 560/590 nm). The same hourly monitoring continued for 30 h after the addition of N-104, N-80/C-25 (negative control), or ampicillin (positive control; BP1760–5; Fisher Scientific) at a final concentration of 100 μM each. Data for each of the 3,884 strains were normalized to untreated with 10% Alamar Blue background subtracted as the ratio of the viability slope during the first 5 hours of N-104 treatment *versus* the slope 5 h previous.

### Imaging flow cytometry

0.5 ml of 10^6^ cfu/ml PA14 cells in 1 mM Na_2_HPO_4_, 0.18 mM KH_2_PO_4_, 0.27 mM KCl, and 13.7 mM NaCl (pH 7.2) were incubated for 1 h at 35 °C with 10 or 100 μM FITC-N-104 (without, or as a positive control with 70% ethanol) or with the negative control FITC-C-95. Cells were subsequently washed in the same buffer, pelleted, treated with 100 μl of 0.25% trypsin EDTA (Gibco, 25200-056) for 5 min at 35 °C to remove surface-attached peptide, further washed and pelleted, resuspended in 100 μl of the same buffer for fixation with an equal volume of 8% formaldehyde (18,814, Polysciences Inc) and then imaged using an Amnis Imagestream X Mark II imaging flow cytometer. 500 to 1000 counts were analyzed per replicate. To ensure the exclusion of debris and out-of-focus cells, Gradient-RMS (average slope spanning three pixels in an image; 0–100) *versus* Area (number of pixels in an image reported in square microns; 1–200) of channel one was plotted for each experiment with more than 85% of the data falling within the cells-in-focus gate. The fluorescence intensity values of FITC-positive cells (Intensity-MC, channel 2) were analyzed using the IDEAS version 6.2 program (Amnis, Seattle WA) and plotted as histograms with a bin number of 100. FITC-positive cells were selected based on histograms generated from the positive control sample.

### Wide-field fluorescence imaging

Janelia Fluor 549-N-104 and -C-95 (each 25 μg) in 20 μl of M9 minimal salts were incubated with ∼3.75 × 10^9^ cfu of pelleted PA14 cells for 1 h at 35 °C, washed four times with filtered 10 mM Na_2_HPO_4_, 1.8 mM KH_2_PO_4_, 2.7 mM KCl, 137 mM NaCl (pH 7.2), and then treated with 5 μM SYTOX Green to assess membrane integrity. Stained cells (1 μl) were immobilized on coverslip supported 1.5% agarose pads for imaging on a custom-built inverted fluorescence microscope, using 1 W/cm^2^ of 561-nm laser light (Genesis MX561 MTM, Coherent) to excite Janelia Fluor 549 Maleimide and 4 mW/cm^2^ of 488-nm laser light (Coherent) to excite SYTOX Green. Emitted light collected using an oil immersion objective (PLSAPO, 60×, 1.4 NA) was passed through a filter set (zt440/488/561rpc, Chroma Technology) to block excitation light with separation of Janelia Fluor 549 Maleimide and SYTOX Green emission using a diochroic beam splitter (T5g0lpxr-uf3, Chroma Technology) as collected on separate sCMOS cameras (ORCA-Flash 4.0 V2, Hamamatsu Photonics). Brightfield images were collected to obtain the total number of cells in each field of view.

### Bacterial ChloroAlkane Penetration Assay (BaCAPA)

The Bacterial ChloroAlkane Penetration Assay was performed as previously described (49). Briefly, mid-log cultures of *E. coli* Lemo21 (DE3) transfected with pET-21 b (+) containing the cyto_Halo or peri_Halo cDNA insert (for cytoplasm *versus* periplasm expression, respectively) were induced for 2 h with 1 mM isopropyl β-D-1-thiogalactopyranoside. Cultures were pelleted, washed three times with 1 mM Na_2_HPO_4_, 0.18 mM KH_2_PO_4_, 0.27 mM KCl, 13.7 mM NaCl (pH 7.2) and then resuspended in the same buffer at a density of ∼4 × 10^9^ cfu/ml. Subsequently, 50 μl aliquots of cells were treated with 0, 4, 8, 16, 32, or 64 μM chloroalkane tagged N-104 (or C-95) for 30 min at 37 °C with agitation, washed with 1 mM Na_2_HPO_4_, 0.18 mM KH_2_PO_4_, 0.27 mM KCl, 13.7 mM NaCl (pH 7.2), exposed to 50 μM of rhodamine chloroalkane (G3221, Promega) for 30 min at 37 °C with shaking, washed with 1 mM Na_2_HPO_4_, 0.18 mM KH_2_PO_4_, 0.27 mM KCl, 13.7 mM NaCl (pH 7.2), fixed for 30 min at room temperature with 2% formaldehyde solution, and then subjected to flow cytometry (Attune NxT Acoustic Focusing Cytometer, Invitrogen) with blue laser excitation (530 nm).

### N-104 affinity purification of bacterial lysate

For generation of N-104 and negative control C-95 affinity columns, 2 mg of Cys(N)-N-104 or Cys(N)-C-95 in 2 ml of 50 mM Tris (pH 8.5), 5 mM EDTA coupling buffer were incubated with an equal volume of suspended SulfoLink Coupling Resin with mixing for 3 h at room temperature. Columns were subsequently washed with 3 ml of coupling buffer followed by 2 ml of freshly prepared 50 mM L-cysteine for 2 h at room temperature for quenching and then washed with 10 ml of 1 M NaCl prior to use or storage in 0.05% sodium azide at 4 °C. Coupling efficiency was determined using fluorescence from N-104's penultimate C-terminal tryptophan as 0.65 ± 0.04 μmole per ml of resin.

Screening for PA14 proteins with N-104 affinity columns was initiated from pelleted 2.5 L overnight cultures that were washed with ice-cold 10 mM Na_2_HPO_4_, 1.8 mM KH_2_PO_4_, 2.7 mM KCl, 137 mM NaCl (pH 7.2) and lysed by sonication in 25 ml of 50 mM Tris (pH 7.5), 1 mM MgCl_2_, 200 mM *n*-octyl-β-D-glucopyranoside with protease inhibitors. Sonication involved five cycles of 15 s with 45 s rest on ice followed by vortexing on ice for 30 min before being rocked end-over-end overnight at 4 °C. After centrifugation, the supernatant was subjected to end-over-end mixing with a 3-ml pre-column of Pierce Agarose followed by equal division by protein concentration for end-over-end incubation with 1 ml N-104 or C-95 columns at 4 °C. Columns were washed with 25 ml of 50 mM Tris (pH 7.5), 50 mM *n*-octyl-β-D-glucopyranoside, 20 mM KCl containing the same protease inhibitors, followed by elution using KCl (50, 75, 100, 125, 150, 300, 500 and 1000 mM) in the same buffer. The 500 mM KCl eluate was subjected to mass spectrometric sequencing by UVA's Biomolecular Analysis Facility. Briefly, eluted samples reduced in 0.1 M NH_4_HCO_3_ with 10 mM dithiolthreitol for 30 min at room temperature were alkylated with 50 mM iodoacetamide in the same buffer (30 min, room temperature), digested with magnetically immobilized trypsin overnight at 37 °C, and then desalted and concentrated on reverse-phase C18 tips. Samples were analyzed with a Thermo Orbitrap Exploris 480 mass spectrometer. In total, approximately 25,000 MS/MS spectra were generated, with ions ranging in abundance over several orders of magnitude. The acquired data were analyzed by database searching using the Sequest search algorithm against UniProt *P. aeruginosa* PA14. N-104 (vs C-95) affinity purification also explored whether there was an affinity for recombinant FeoB and PotH, and for other tear bactericidal proteins (see below).

### FeoB and PotH recombinant expression and purification

We used the pFeoB plasmid (6) with *feoB* insert from *P. aeruginosa* PAO1 in pET41-a(+) with a C-terminal 8-His tag and kanamycin-resistant cassette, and *E. coli* EcCD00397460 plasmid (DNASU Plasmid Repository, Tempe Arizona) with *potH* insert in PCDF Bravo with C-terminal 10-His tag and streptomycin-resistant cassette. Plasmids of each were generated using standard procedures. His-tagged FeoB or PotH proteins in the supernatant were captured on Ni-Sepharose for 4 h with end-over-end rotation at 4 °C, washed with 10 mM HEPES potassium salt, 10% glycerol, 0.5 M NaCl, 10 mM MgSO_4_, 10 mM imidazole, and 0.05% dodecyl-β-D-maltopyranoside and eluted with the same wash buffer but containing 0.2 M NaCl and 0.5 M imidazole. Eluted FeoB and PotH were concentrated into 10 mM HEPES potassium salt, 10% glycerol, 10 mM MgSO_4_, 0.05% dodecyl-β-D-maltopyranoside and 0.15 M NaCl respectively on Amicon Ultra-0.5 Centrifugal UFC505024, UFC503024 and UFC900596 filter units.

### FeoB and PotH ligation of N-104

N-104 and C-95 columns (1 ml each) equilibrated in 10 mM HEPES potassium salt, 10% glycerol, 10 mM MgSO_4_, 0.05% dodecyl-β-D-maltopyranoside, and 0.15 M NaCl were individually mixed at 4 °C overnight with purified FeoB and PotH. After collecting the flowthrough, columns were washed with 25 ml of 10 mM HEPES, 0.05% dodecyl-β-D-maltopyranoside, and 0.15 M KCl, and were subjected to stepwise elution using 1 ml of the same buffer containing progressively increasing concentrations of KCl (0.3, 0.5, and 1.0 M). Equal volumes of the flow through, final wash fraction and KCl elution fractions were then denatured in 4× Laemmli buffer containing 10% β-mercaptoethanol at room temperature for 15 to 30 min without boiling to avoid aggregation, separated by SDS-PAGE on 4 to 20% gradient gels, transferred to nitrocellulose, blocked with 1% fish gelatin in 10 mM Na_2_HPO_4_, 1.8 mM KH_2_PO_4_, 2.7 mM KCl, 137 mM NaCl (pH 7.2) supplemented with 0.1% Tween-20, and incubated overnight (4 °C) with 10 ml of 1 μg/ml of rabbit anti-His tag. After three washes in 10 mM Na_2_HPO_4_, 1.8 mM KH_2_PO_4_, 2.7 mM KCl, and 137 mM NaCl (pH 7.2) supplemented with 0.1% Tween-20, blots were incubated with 0.1 μg/ml of IRDye 680RD labeled donkey antirabbit IgG at room temperature for 75 min. Primary and secondary antibodies were diluted in 10 mM Na_2_HPO_4_, 1.8 mM KH_2_PO_4_, 2.7 mM KCl, 137 mM NaCl (pH 7.2) containing 0.1% Tween-20 and 0.05% sodium azide with LI-COR Odyssey CLx detection.

In other experiments, 1 ml of ∼160 and 80 μg, respectively, each of FeoB and PotH incubated with 200 μl N-104 column aliquots (final FeoB and PotH concentrations of ∼2.5 μM) were subjected to increasing concentrations (0, 1, 10, 100 or 250 μM) of FeoB outer loop two or five synthetic peptides, SDC1 synthetic peptide 19 to 30, or PotH outer loop synthetic peptides two or three with end-over-end rocking overnight at 4 °C. DMSO was used as the solvent for FeoB outer loop 2, and PotH outer loop 2 and 3. Water was used as the solvent for FeoB outer loop five and SDC1 (19–30). After flowthrough collection, columns were washed with 5 ml of 10 mM HEPES, 0.05% dodecyl-β-D-maltopyranoside, 0.15 M KCl and then were subjected to elution with 200 μl of the same buffer containing 1.0 M KCl. 1.0 M elutions were separated by SDS-PAGE and Western blotting as per above.

### Iron transport assay

Iron depletion was induced in PA14 cells (5 × 10^9^ cfu in 10 ml 10 mM Na_2_HPO_4_, 1.8 mM KH_2_PO_4_, 2.7 mM KCl, 137 mM NaCl (pH 7.2) by treatment with the chelator 1 mM 2,2′-bipyridyl at room temperature for 1 h. After washing twice with 0.9% NaCl, cells were suspended in 1 ml of 1 mM Na_2_HPO_4_, 0.18 mM KH_2_PO_4_, 0.27 mM KCl, 13.7 mM NaCl for treatment without or with 1, 10, 25, 50 or 100 μM of N-104 (or negative control N-80/C-25 or C-95) for 45 min at 35 °C, incubated at the same temperature for 15 min with 100 μM FeSO_4_ and 13 mM ascorbic acid, washed twice with 1 mM Na_2_HPO_4_, 0.18 mM KH_2_PO_4_, 0.27 mM KCl, and 13.7 mM NaCl, and pelleted at 12,000*g*. The ferrozine assay (54) was subsequently employed. Briefly, cells in pellets were lysed with 20 μl of 50 mM NaOH, partially neutralized with 20 μl of 10 mM HCl, subjected to iron release with 20 μl of acidified 0.29 M KMnO_4_ for 30 min at 80 °C, cooled to room temperature, incubated with 7 μl of 30 mM ferrozine, 1.1 μl of 180 mM neocuproine, 15 μl of 5 M ammonium acetate and 15 μl of 1 M ascorbic acid for 30 min at room temperature and then centrifuged at 21,000*g* for 10 min. Cellular iron uptake could then be assessed from the supernatant OD_565_. Single-molecule transport per cell was estimated using a standard curve.

### Polyamine transport assay

100 μl aliquots (0.8 × 10^8^ cfu) of PA14 cell cultures in 1 mM Na_2_HPO_4_, 0.18 mM KH_2_PO_4_, 0.27 mM KCl, 13.7 mM NaCl without or with 1, 10, 25, 50 or 100 μM N-104 (or N-80/C-25 or C-95) for 45 min at 35 °C were incubated with 1 μl of 10 mM [1,4-^14^C]putrescine (0.1 mCi/ml; ARC-0245–250, American Radiolabeled Chemicals) or [1,4-^14^C]spermidine (0.1 mCi/ml; ARC-3138–50, American Radiolabeled Chemicals) for 15 min at 35 °C. Cell pellets were washed four times with 100 μl of 10 mM Na_2_HPO_4_, 1.8 mM KH_2_PO_4_, 2.7 mM KCl, 137 mM NaCl (pH 7.2) and then transferred to 4 ml of scintillation fluid (1200.437, PerkinElmer OptiPhase HiSafe 3) for analysis in a Beckman Coulter LS6500. Also assessed was radioactivity from the last wash. Single-molecule transportation per cell was estimated using standard curves for each radiolabeled polyamine.

### Screening for proteins or peptides in human tears that are synergistic with N-104

Studies in this work abide by the Declaration of Helsinki principles. As per UVA IRB protocol 18,592, consented normal human volunteers were subjected to 0.5% proparacaine anesthesia of the eye to minimize reflex tearing. Human basal tears were then wicked for 5 min onto filter paper-like Schirmer strips. Those that had wicked a distance of at least 12 mm were subjected to 4 °C elution with 10 mM Na_2_HPO_4_, 1.8 mM KH_2_PO_4_, 2.7 mM KCl, 137 mM NaCl (pH 7.2) for a final total pooled tear volume of 170 μl. The pool was diluted five-fold in 10 mM Na_2_HPO_4_, 1.8 mM KH_2_PO_4_, 2.7 mM KCl, and 137 mM NaCl (pH 7.2) with protease inhibitors and subjected to end-over-end mixing with a 100 μl precolumn of Pierce Agarose followed by equal division by protein concentration into each of 100 μl of N-104 or C-95 columns. The solution was mixed for 5 h at 4 °C. Columns were subjected to washing with 10 mM Na_2_HPO_4_, 1.8 mM KH_2_PO_4_, 2.7 mM KCl, and 137 mM NaCl (pH 7.2) containing the same protease inhibitors followed by elution using KCl (0.3, 0.5, and 1.0 M KCl) in the same buffer. The 0.5 M KCl eluate was submitted for mass spectrometric sequencing by UVA's Biomolecular Analysis Facility, as previously noted. The acquired data were analyzed by database searching using the Sequest search algorithm against UniProt *H. sapiens*. Anti-thrombin western blotting of non-boiled flowthrough, final wash fraction, and KCl elution fractions in 4× Laemmli buffer containing 10% β-mercaptoethanol was performed after separation on 4 to 20% gradient SDS-PAGE gels. Proteins were transferred onto nitrocellulose membranes, blocked with 1% fish gelatin in 10 mM Na_2_HPO_4_, 1.8 mM KH_2_PO_4_, 2.7 mM KCl, 137 mM NaCl (pH 7.2) supplemented with 0.1% Tween-20 and then incubated overnight (4 °C) with 10 ml of 1 μg/ml of polyclonal rabbit anti-coagulation factor II/thrombin antibody in 10 mM Na_2_HPO_4_, 1.8 mM KH_2_PO_4_, 2.7 mM KCl, 137 mM NaCl (pH 7.2) supplemented with 0.1% Tween-20 and 0.05% sodium azide. After three washes in 10 mM Na_2_HPO_4_, 1.8 mM KH_2_PO_4_, 2.7 mM KCl, and 137 mM NaCl (pH 7.2) supplemented with 0.1% Tween-20, blots were incubated with 0.1 μg/ml of IRDye 680RD labeled donkey antirabbit IgG at room temperature for 75 min. Primary and secondary antibodies were diluted in 10 mM Na_2_HPO_4_, 1.8 mM KH_2_PO_4_, 2.7 mM KCl, and 137 mM NaCl (pH 7.2) containing 0.1% Tween-20 and 0.05% sodium azide. After washing in the same way, imaging was performed on a LI-COR Odyssey CLx.

Synthesized peptides corresponding to identified and selected N-104 binding proteins were subjected to modified PA14 checkerboard killing assays with N-104. Sterile microtiter wells containing 25 μl of N-104 serially diluted along the ordinate and an equal volume of candidate synthetic peptide serially diluted along the abscissa were incubated aerobically with 100 μl of an overnight culture of PA14 at 10^6^ cfu/ml in 10 mM Na_2_HPO_4_, 1.8 mM KH_2_PO_4_, 2.7 mM KCl, 137 mM NaCl (pH 7.2) at 35 °C for 7 to 8 h after which 150 μl of LB broth was added to each well for an additional 16 to 18 h of incubation (35 °C). Well-mixed 2 μl aliquots of each were then dropped onto LB plates for overnight incubation and imaging.

The interaction of N-104 with synergistic thrombin peptide GKY20 was explored by drying 1, 5, and 20 μg of GKY20 onto nitrocellulose, blocking with 1% fish gelatin in 10 mM Na_2_HPO_4_, 1.8 mM KH_2_PO_4_, 2.7 mM KCl, 137 mM NaCl (pH 7.2) for 1 h at room temperature, and then adding 10 ml of 20 μM N-104 or of each N-104 analog, or of negative control C-95, for 2 h at 35 °C with shaking. After washing three times for 10 min, each with 10 ml of 10 mM Na_2_HPO_4_, 1.8 mM KH_2_PO_4_, 2.7 mM KCl, 137 mM NaCl (pH 7.2), nitrocellulose membranes were incubated overnight at 4 °C with 1 μg/ml of either rabbit polyclonal ab-C-term with specificity for the C-terminal 54 amino acids of lacritin (86) appropriate for N-104 or its analogs, or with rabbit anti-lacritin N-terminus antibody ab-N-term appropriate for C-95. Membranes washed three times for 10 min each with 10 ml of 10 mM Na_2_HPO_4_, 1.8 mM KH_2_PO_4_, 2.7 mM KCl, 137 mM NaCl (pH 7.2), were then incubated with 0.1 μg/ml of IRDye 680RD labeled donkey antirabbit IgG (926-68073, LI-COR) as described above for LI-COR Odyssey CLx detection.

### Murine corneal infection assay

*In vivo* PA14 infection studies were performed using the murine corneal scratch-injury protocol as previously reported (63). Briefly, 18-h aerobic (37 °C) PA14 cultures were prepared from a stock vial inoculated onto tryptic soy agar plates with 5% sheep blood. Bacterial cells were suspended in 1% proteose peptone to an OD_650_ nm of 1.0 and diluted to ∼1 to 1.2 × 10^6^ cfu in 5 μl to prepare the inoculum for ocular infections. The actual inoculum was confirmed by dilution in 1% proteose peptone containing 0.05% Triton X-100 for bacterial enumeration on agar plates. 30 C57/BL6 mice (7 weeks old; female) anesthetized by intraperitoneal injection of ketamine (100 mg/kg) and xylazine (10 mg/kg), as confirmed by the absence of a toe pinch response, were each subjected to three 26-gauge needle scratches running from 3 to 5 mm along the length of one cornea followed by placement of 5 μl of PA14 inoculum. Four and 8 h later, 5 μl of a mixture of 1 μM of GKY20 and 20 μM N-104 in 10 mM Na_2_HPO_4_, 1.8 mM KH_2_PO_4_, 2.7 mM KCl, 137 mM NaCl (pH 7.2) was applied to the infected eye in anesthetized mice. After 24 h, eyes were assigned a 0 to 4 pathology score using the following scheme: 0, eye macroscopically identical to the uninfected contralateral control eye; 1, faint opacity partially covering the pupil; 2, dense opacity covering the pupil; 3, dense opacity covering the entire anterior segment; and 4, perforation of the cornea and/or phthisis bulbi (shrinkage of the globe after inflammatory disease). Mice were euthanized by an overdose of CO_2_ followed by cervical dislocation. Corneas dissected off from enucleated whole eyes were then homogenized in 500 ul of 1% proteose peptone containing 0.05% Triton X-100 for serial dilution and bacterial plating to estimate bacterial CFU infectivity. All procedures were carried out in accordance with the Association for Research in Vision and Ophthalmology resolution on the use of animals in research and were approved by the Brigham and Women’s Hospital Institutional Animal Care and Use Committee, protocol 2018N000003.

### RNAseq of N-104 treated PA14

For RNA extraction and sequencing of *P. aeruginosa*, we used diethylpyrocarbonate (DEPC) treated Eppendorf tubes (except for those used for synthetic peptide incubation). An aliquot of PA14 overnight culture grown for 3.5 to 4.0 h in LB broth to OD_600_ = 0.5 (5.0 × 10^8^ cells) was gently pelleted (12,000*g*, 30 s), resuspended in 0.5 ml of 1 mM Na_2_HPO_4_, 0.18 mM KH_2_PO_4_, 0.27 mM KCl, 13.7 mM NaCl (pH 7.2) and incubated with five or 20 μM N-104, or 20 μM N-80/C-25 for 45 min at 35 °C or left untreated. After pelleting (12,000*g*, 5 min), cells were resuspended in 0.5 ml of TE buffer supplemented with 1 ml of RNA-protect Bacteria Reagent with 5 s of vortexing prior to incubation for 5 min at room temperature, pelleting (5000*g*, 10 min), and lysis initiated in 200 μl of 15 mg/ml lysozyme together with 20 μl of 2.5 mg/ml proteinase K, both in TE buffer. Vortexing and agitation were followed by the addition of 20 μl of 10% SDS in DEPC-treated water (74104, Qiagen) according to the manufacturer's directions. The resulting RNA samples were stored at −80 °C. By gel electrophoresis, two sharp 18s and 28s rRNA peaks were apparent with an RNA Integrity Number equivalent (RINe) of 8.4 to 9.2 and a concentration of approximately 40 to 60 ng/μl. Library construction and sequencing were performed by UVA's Genome Analysis and Technology Core involving mRNA enrichment *via* the NEBNext rRNA Depletion Kit (Bacteria), and transcriptome sequencing on a Next Seq 500 Mid Output platform, with 150-bp paired-end reads. The range of raw reads for each sample varied from 8 to 10 million, thus ensuring an optimal depth of sequencing that was analyzed by UVA's Bioinformatics Core facility.

### Quantification and statistical analysis

A minimum of three biological replicates were performed for all experiments, unless explicitly specified otherwise as noted in the Figure Legends. All data are presented as the means ± SDs, with single data points represented. After an initial Shapiro-Wilk normality test, significance was largely evaluated using the Friedman test if nonparametric or by one- and two-way ANOVA with multiple comparisons tests (Prism), as documented in Figure Legends. Significant differences were noted as ∗∗∗∗*p* < 0.0001, ∗∗∗*p* < 0.001, ∗∗*p* < 0.01, and ∗*p* < 0.05.

## Data availability

Addgene plasmid numbers for all unique recombinant DNA generated in this study are documented in the Supporting Information Reagents Table. These and all other unique reagents generated in this study are available upon request. Tandem mass spectrometry data are available in the MassIVE Repository as: MassIVE MSV000094085 (https://massive.ucsd.edu/ProteoSAFe/dataset.jsp?task=27f1ca8b86734a2bad287e523e2103ee) and MassIVE MSV000094086 (https://massive.ucsd.edu/ProteoSAFe/dataset.jsp?task=72147a3716e34e7eb2c24f084ee08436). RNAseq data are available in NCBI GEO DataSets: Accession ID: GSE253123. All other data are available in the manuscript and in Supporting Information Figures.

## Supporting information

This article contains supporting information.

## Conflict of interest

The authors declare the following financial interests/personal relationships which may be considered as potential competing interests: G. W. L. is cofounder and C. S. O. of TearSolutions, Inc; and cofounder and C. T. O. of IsletRegen, LLC. Other authors declare no competing interests.
