# Supplementary material for: Lacritin cleavage-potentiated targeting of iron - respiratory reciprocity promotes bacterial death
Source: J Biol Chem. 2025 Mar 26;301(5):108455. doi: 10.1016/j.jbc.2025.108455 (PMC12147183; doi:10.1016/j.jbc.2025.108455)
Supplement: Supporting Information [file mmc1.docx]

Supporting Information

**Lacritin Cleavage-Potentiated Targeting of Iron - Respiratory Reciprocity Promotes Bacterial Death**

Mohammad Sharifian Gh., Fatemeh Norouzi, Mirco Sorci, Tanweer S. Zaidi, Gerald B. Pier, Alecia Achimovich, George M. Ongwae, Binyong Liang, Margaret Ryan, Michael Lemke, Georges Belfort, Mihaela Gadjeva, Andreas Gahlmann, Marcos M. Pires, Henrietta Venter, Thurl E. Harris and Gordon W. Laurie

Corresponding authors: Mohammad Sharifian Gh.: sharifian@virginia.edu

Gordon Laurie: glaurie@virginia.edu

**The PDF file includes:**

Supplementary Figures 1 - 7

Resources Table

Supplementary Tables 1 - 3

Source Data F4, F6

**
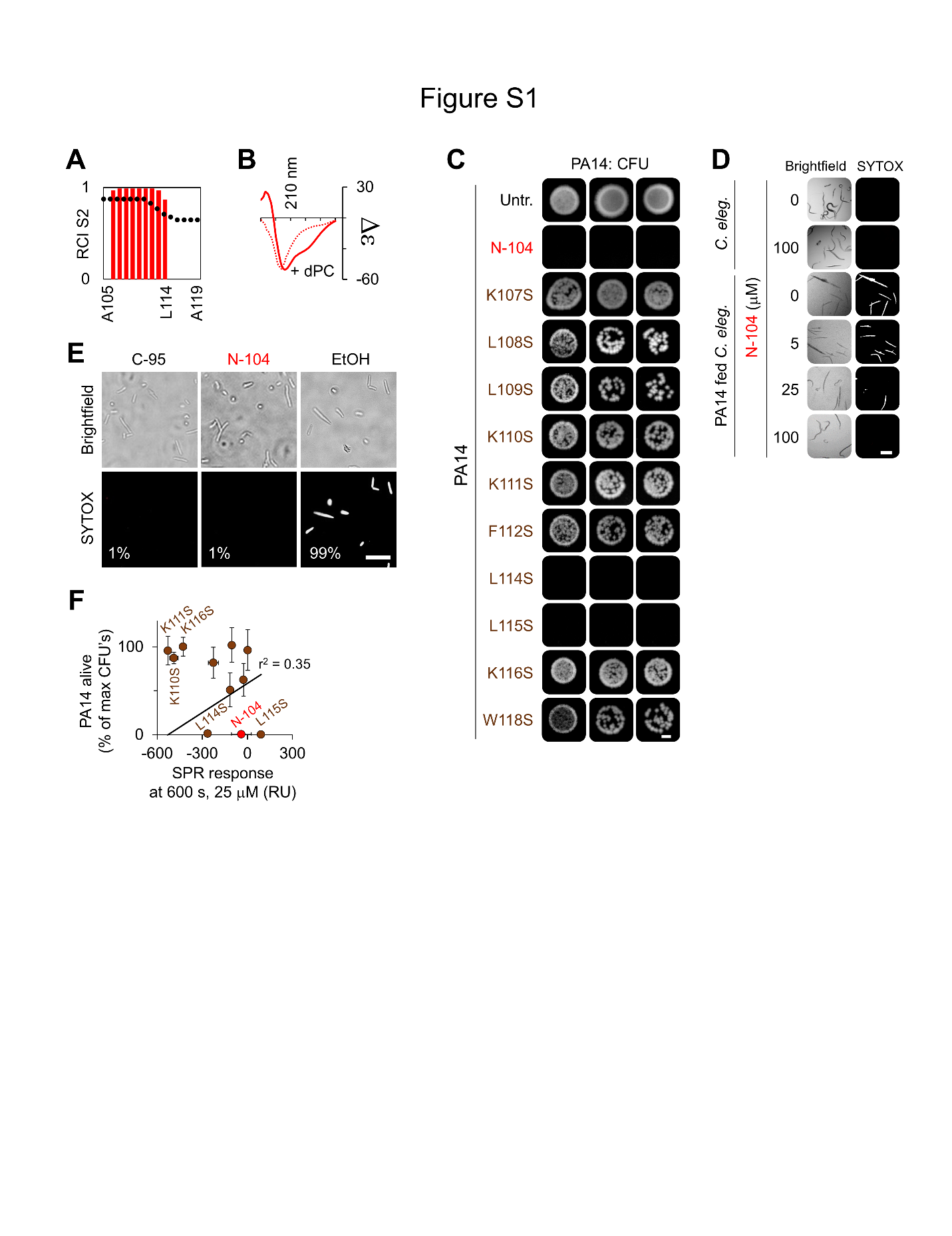
**

**Supplementary Fig. 1. Lysis-free 'N-104' killing of virulent and multi-drug resistant *P. aeruginosa* strain 'PA14' together with biological replicates related to Fig. 1.** *A*, ^1^H-NMR predicted α-helicity (red bars) and random coil index S2 (RCI S2; black dots) values of 1 mM N-104 in phosphate buffer containing 150 mM dodecylphosphocholine. *B*, Circular dichroism of N-104 in phosphate buffer in the presence (solid line) or absence (dashed line) of 10 mM dodecylphosphocholine ('dPC') over a 190-240 nm range. *C*, Biological replicates of Fig. 1C CFU assay wherein overnight cultures of PA14 (10^6^ cfu/ml) were were incubated in suspension for 8 hrs without (untreated; ‘Untr’) or with 25 µM N-104 or each N-104 analog and then plated onto LB agar. Scale bar is 2 mm. *D*, Optimization study for Fig. 1D *C. elegans* survival assay in which overnight cultures of PA14 (10^7^ cfu/ml) treated for 5 hrs with 0 - 100 µM N-104 were fed to 50 *C. elegans* worms per well in 96-well plates for three days. After removal of bacteria, an equal volume of SYTOX Orange was added to fluorescently image dead worms. Scale bar is 200 μm. *E*, PA14 membrane disruption assay after incubation of overnight cultures (10^7^ cfu/ml) for one hour at 35ºC with 25 µM N104 or C-95 or with positive control 70% ethanol. Detection is with 5 µM SYTOX orange. Scale bar is 200 μm. *F*, Linear regression of PA14 viability versus the SPR response at 600 s on chip supported PC:PG (75:25) of 25 µM N-104 or N-104 analogs ([mean with S.D.] n = 4).


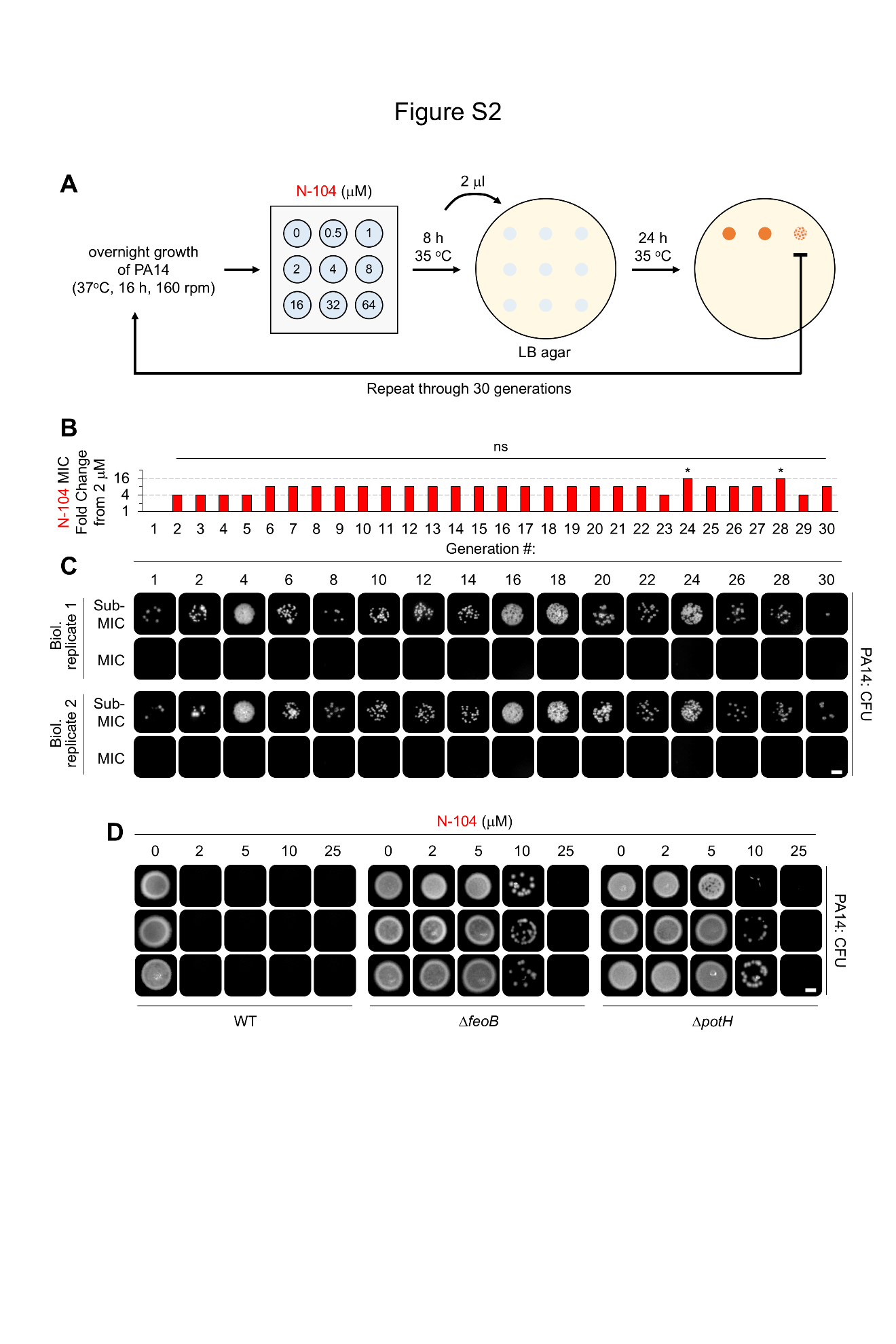


**Supplementary Fig. 2. Thirty generations of PA14 fail to develop stable N-104 resistance together with biological replicates related to Fig. 2.**  *A*, Schematic of the N-104 sub-minimal inhibitory concentration (‘sub-MIC’) N-104 resistant assay. Pelleted overnight cultures of PA14 (10^6^ cfu/ml) in 100 µl were incubated with 0 - 64 µM N-104 for 8 hour at 35ºC and then plated onto LB agar. Sub-MIC colonies (10^6^ cfu/ml) in 100 µl were selected and incubated with 0 - 64 µM N-104 for 8 hour at 35ºC and then plated onto LB agar. This process was repeated through 30 generations. *B*, Generational fold change of N-104 MIC over 30 generations. The original sub-MIC N-104 concentration was 2 µM, for which there was no initial sub-MIC change ([mean], n = 2, *p<0.05; ns, not significant [Friedman ANOVA with Dunn's multiple comparisons test]). *C*, Biological replicates of the N-104 sub-MIC resistant assay. Scale bar is 2 mm. *D*, Biological replicates of the Fig. 2F CFU assay wherein overnight cultures of PA14 wild type (WT), or Δ*feoB* or Δ*potH* transposon mutants PA14 were treated in suspension for 8 hrs with 0 - 25 µM N-104 and then plated onto LB agar. Scale bar is 2 mm.

**
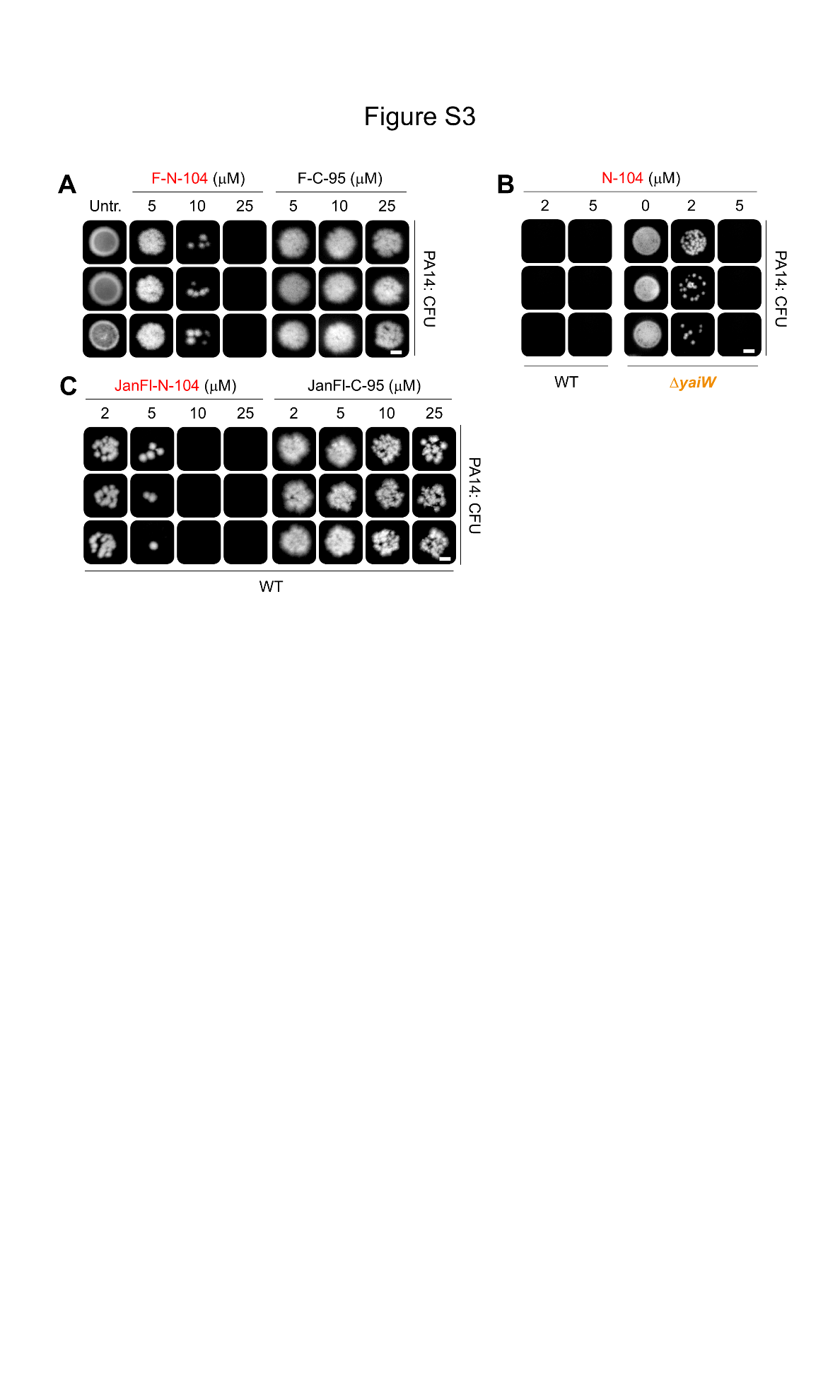
**

**Supplementary Fig. 3. N-104 activity is partially affected by FITC and Janelia Fluor 549 tagging together with biological replicates related to Fig. 3.** *A*, CFU assay wherein overnight cultures of PA14 (10^6^ cfu/ml) were incubated in suspension for 8 hrs with 0 - 25 µM FITC-N-104 (‘F-N-104’) or 5 - 25 µM FITC-C-95 (‘F-C-95’) and then plated onto LB agar. Scale bar is 2 mm. The same approach was utilized in *B* and *C*. *B*, PA14 CFU assay in which wild type PA14 (WT) and PA14 Δ*yaiW* (both 10^6^ cfu/ml) were respectively with 2, 5 and 0 - 5 µM N-104 followed by plating on LB agar. *C*, CFU assay of PA14 (10^6^ cfu/ml) incubated with 2 - 25 µM Janelia Fluor 549-tagged N-104 or Janelia Fluor 549-tagged C-95 for 8 hrs and then plated onto LB agar. Scale bar is 2 mm.


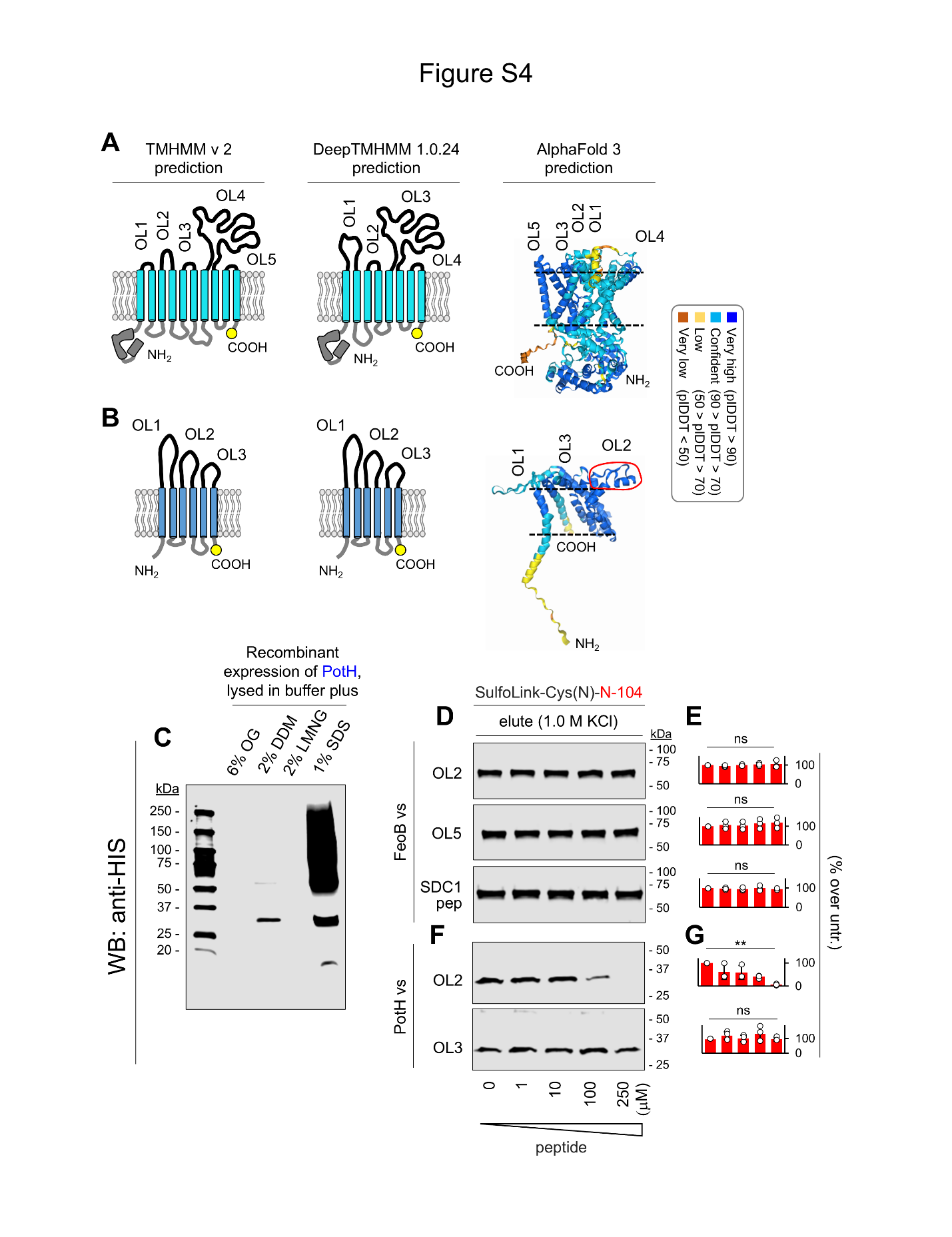


**Supplementary Fig. 4. Models of FeoB and PotH together with outer loop peptides competition assay related to Fig. 4.** *A*, TMHMM v 2, Deep TMHMM 1.0.24, and AlphaFold 3 predicted FeoB models with periplasmic outer loops ('OL'). FeoB HIS-tag (yellow circle) is located on the cytoplasmic C-terminus. *B*, TMHMM v 2, Deep TMHMM 1.0.24, and AlphaFold 3 predicted PotH models with periplasmic outer loops. PotH HIS-tag (yellow circle) is located on the cytoplasmic C-terminus. *C*, Recombinant expression of PotH, lysed in buffer containing 6% n-octyl-β-D-glucopyranoside (OG), 2% dodecyl-β-D-maltopyranoside (DDM), 2% lauryl maltose neopentyl glycol (LMNG) or 1% sodium dodecyl sulfate (SDS). *D,F*, His-tagged PotH binding assay with SulfoLinkCys(N)-N-104 columns. Overnight cultures of PA14 overexpressing His-tagged PotH were lysed in buffered 2% DDM with protease inhibitors, captured on nickel columns, eluted, subjected to a buffer change and then passed onto a SulfoLinkCys(N)-N-104 column at 4ºC in which binding was performed in the presence of synthetic peptides corresponding to PotH outer loop 2 or 3 or FeoB outer loop 2 or 5 or SDC1 synthetic peptide 19-30 each at 0 - 250 µM as potential soluble inhibitors. Shown is the flowthrough (flowthr), last wash fraction and 0.3 – 1 M KCl elution fractions as detected by anti-His Western blotting. *E,G*, LI-COR quantitation of *M* ([mean with S.D.], n = 3, **p<0.01; ns, not significant [Kruskal-Wallis ANOVA]).


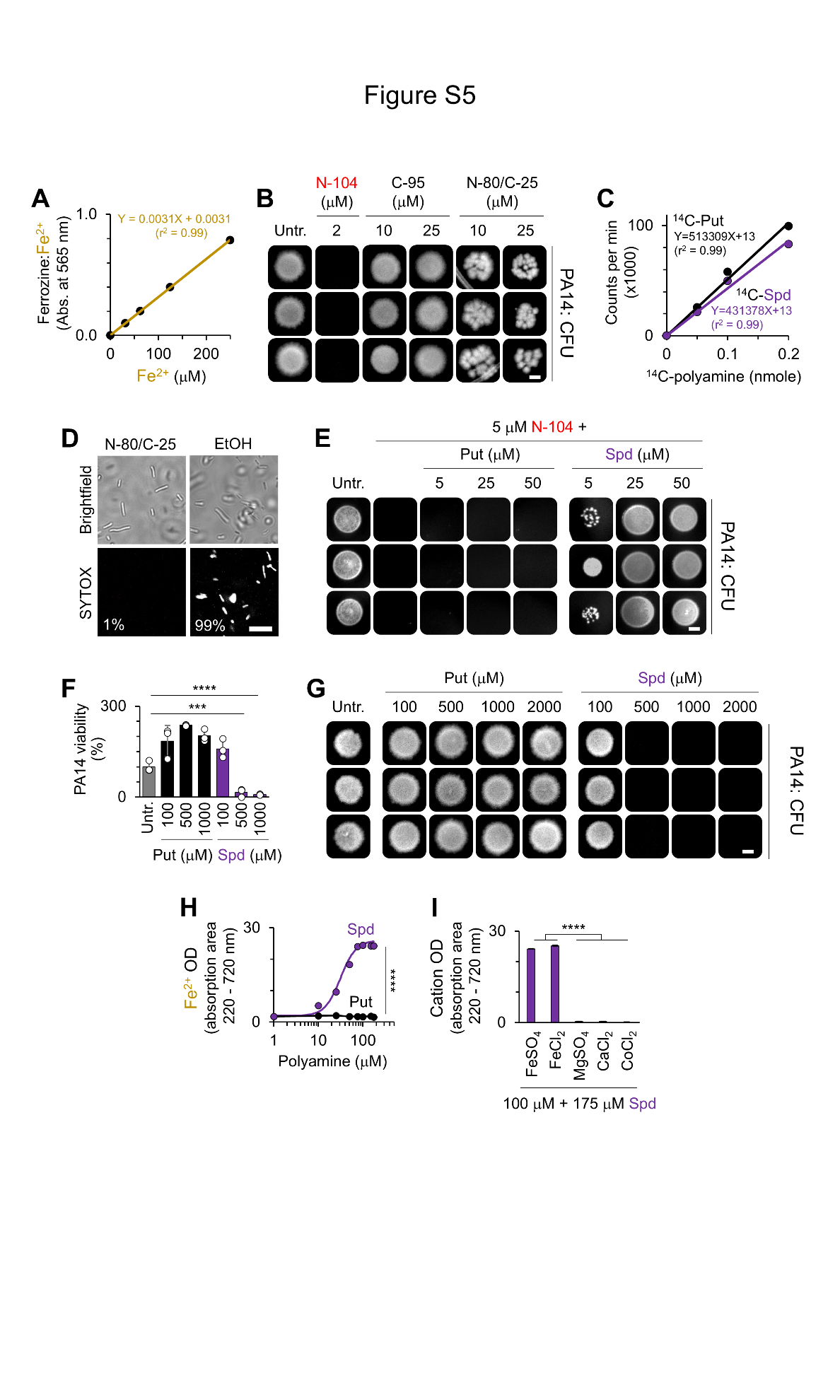


**Supplementary Fig. 5. Biological replicates and supporting data related to Fig. 5.** *A*, Linear regression of colorimetric ferrozine Fe^2+^ detection versus Fe^2+^ concentration for estimation of Fig. 5B - D intracellular iron. 0 - 256 μM FeSO_4_ in 13 mM ascorbic acid was incubated with a mixture of ferrozine, neocurproine, ammonium acetate and ascorbic acid for 30 min. The OD_565_ was then determined. *B*, CFU assay wherein overnight cultures of PA14 (10^6^ cfu/ml) were incubated in suspension for 8 hrs with 0, 2 µM N-104 or 10, 25 µM C-95 or 10, 25 M N-80/C-25 and then plated onto LB agar. Scale bar is 2 mm. The same approach was utilized in *B*, *E and G*. *C*, Linear regression of counts per minute versus increasing concentrations of ^14^C-putrescine (^14^C-Put) and ^14^C-spermidine (^14^C-Spd) for calculation of molar uptake in Fig. 5F - H. ^14^C-putrescine and -spermidine were detected by scintillation counting. *D*, PA14 membrane disruption assay after incubation of overnight cultures (10^7^ cfu/ml) for one hour at 35ºC with 25 µM N-80/C-25 or with positive control 70% ethanol. Detection was with 5 µM SYTOX orange. Scale bar is 200 μm. *E*, Biological replicates of Fig. 5J CFU assay wherein overnight cultures of PA14 (10^6^ cfu/ml) were either untreated or treated in suspension with 5 µM N-104 in the absence of presence of 5 - 50 µM putrescine or spermidine for eight hours and then applied to LB agar for overnight growth. Scale bar is 2 mm. *F*, Viability asay in which overnight cultures of PA14 (10^6^ cfu/ml) were untreated or treated with 100 - 1000 µM putrescine or spermidine followed by 10% Alamar Blue viability monitoring overnight at 37ºC ([mean with S.D.], n = 3, ****p<0.0001, ***p<0.001 [Two-way ANOVA with uncorrected Fisher's least significant difference test]). *G*, CFU assay in which overnight cultures of PA14 (10^6^ cfu/ml) were either untreated or treated with 100 - 2000 µM putrescine or spermidine at 37ºC prior to application to LB agar. Scale bar is 2 mm. *H*, Fe^2+^ binding assay in the presence of 1 – 175 µM putrescine or spermidine. Putrescine and spermidine were added to 100 µM FeSO_4_ for 15 min. Fe^2+^ associated absorbance (OD 220-720 nm) was then monitored ([mean with S.D.], n = 3, ****p<0.0001 [Two-way ANOVA]). *I*, Comparative Fe^2+^, Fe^3+^, Mg^2+^, Ca^2+^, Co^2+^ binding assay in the presence of 175 µM spermidine. 100 µM of each as salts was added. Cation absorbance (OD 220-720 nm) was then monitored. ([mean with S.D.], n = 3, ****p<0.0001 [One-way ANOVA with Tukey's multiple comparisons test]).


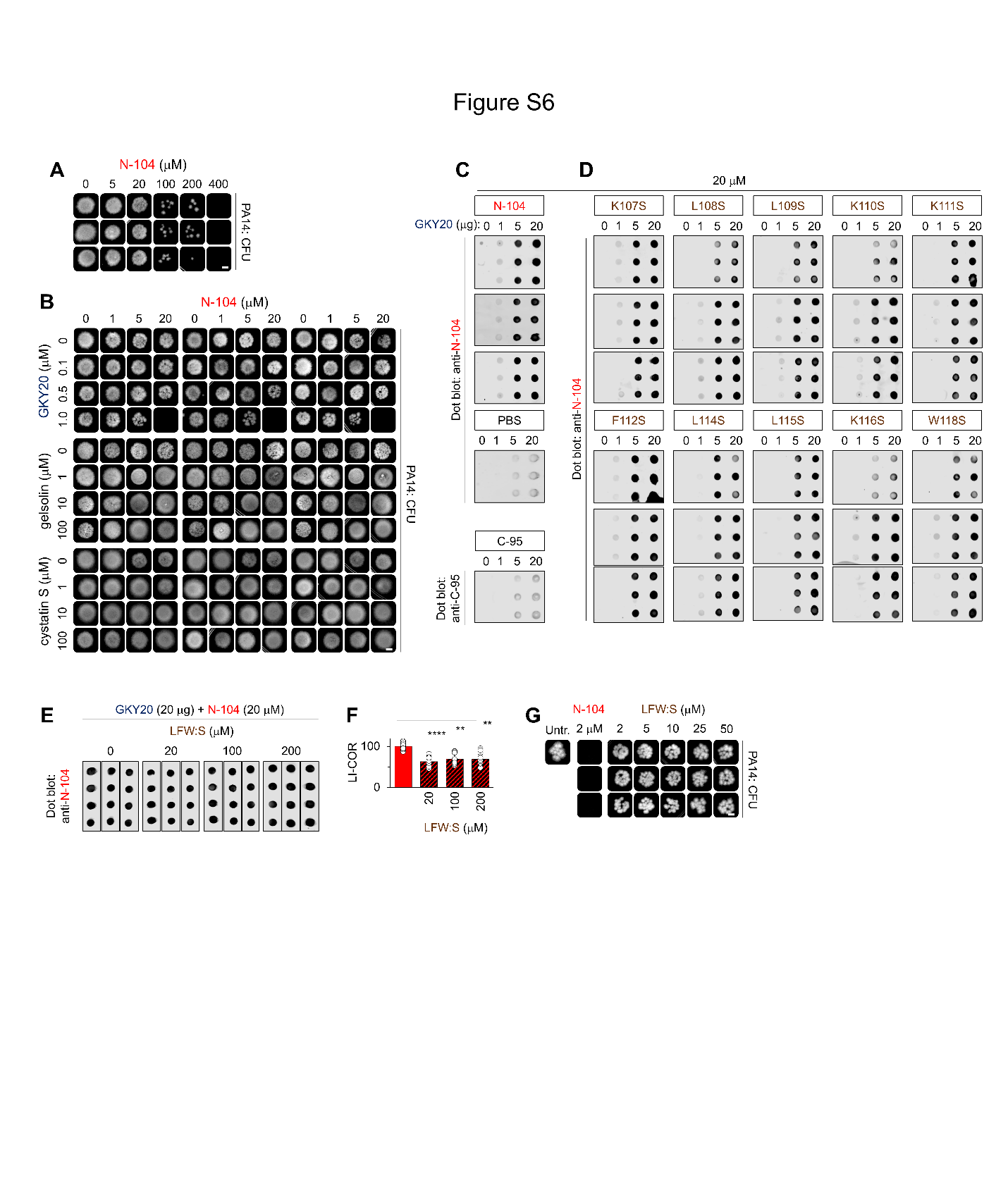


**Supplementary Fig. 6. N-104 efficacy at 300 mOsm/l and synergy with thrombin GKY20 synthetic peptide, together with biological replicates related to Fig. 6.** *A*, Biological replicates of the Fig. 6A CFU assay (300 mOsm/l portion) whereby overnight cultures of PA14 (10^6^ cfu/ml) were incubated in suspension for 8 hrs with 0 - 400 µM N-104 in 300 mOsm/l phosphate buffer and then plated onto LB agar. Scale bar is 2 mm. The same method was used in *B* and *G*. *B*, Biological replicates of Fig. 6D PA14 CFU checkerboard assays using the same method as in *A* wherein PA14 was incubated with mixtures of 0 - 20 µM N-104 and 0 - 1 µM GKY20, or 0 - 20 µM N-104 and 0 – 100 µM gelsolin, or 0 - 20 µM N-104 and 0 – 100 µM cystatin S in 300 mOsm/l phosphate buffer and then plated onto LB agar. Scale bar is 2 mm. *C,* Biological replicates of Fig. 6E dot blot binding assay in which 0 - 20 μg of GKY20 was immobilized on nitrocellulose, blocked with 1% fish gelatin, and then incubated with 20 μM N-104 or C-95 for 2 hours at 35^o^C for respective anti-N-104 or anti-C-95 LI-COR detection. *D*, Replicates of the same assay in which N-104 was replaced with each of ten N-104 analog. *E*, Replicates of the binding assay in which 20 µg of GKY20 was immobilized, blocked and then incubated with 20 μM of N-104 along with 0 - 200 μM of N-104 ‘LFW:S’ analog for 2 hours at 35^o^C, for anti-N-104 detection. Since ab-C-term antibody cannot detect N-104 LFW:S, the capacity of increasing amounts of LFW:S to inhibit N-104 ligation of GKY20 was assessed. *F*, LI-COR quantitation of *E* ([mean with S.D.], n = 9, ****p<0.0001, **p<0.01 [Friedman ANOVA with Dunn's multiple comparisons test]). *G*, PA14 colony forming unit (CFU) assay whereby overnight cultures of PA14 (10^6^ cfu/ml) were incubated in suspension for 8 hrs without or with 2 μM N-104 or 2, 5, 10, 25 or 50 µM N-104 LFW:S, and then plated onto LB agar. Scale bar is 2 mm.


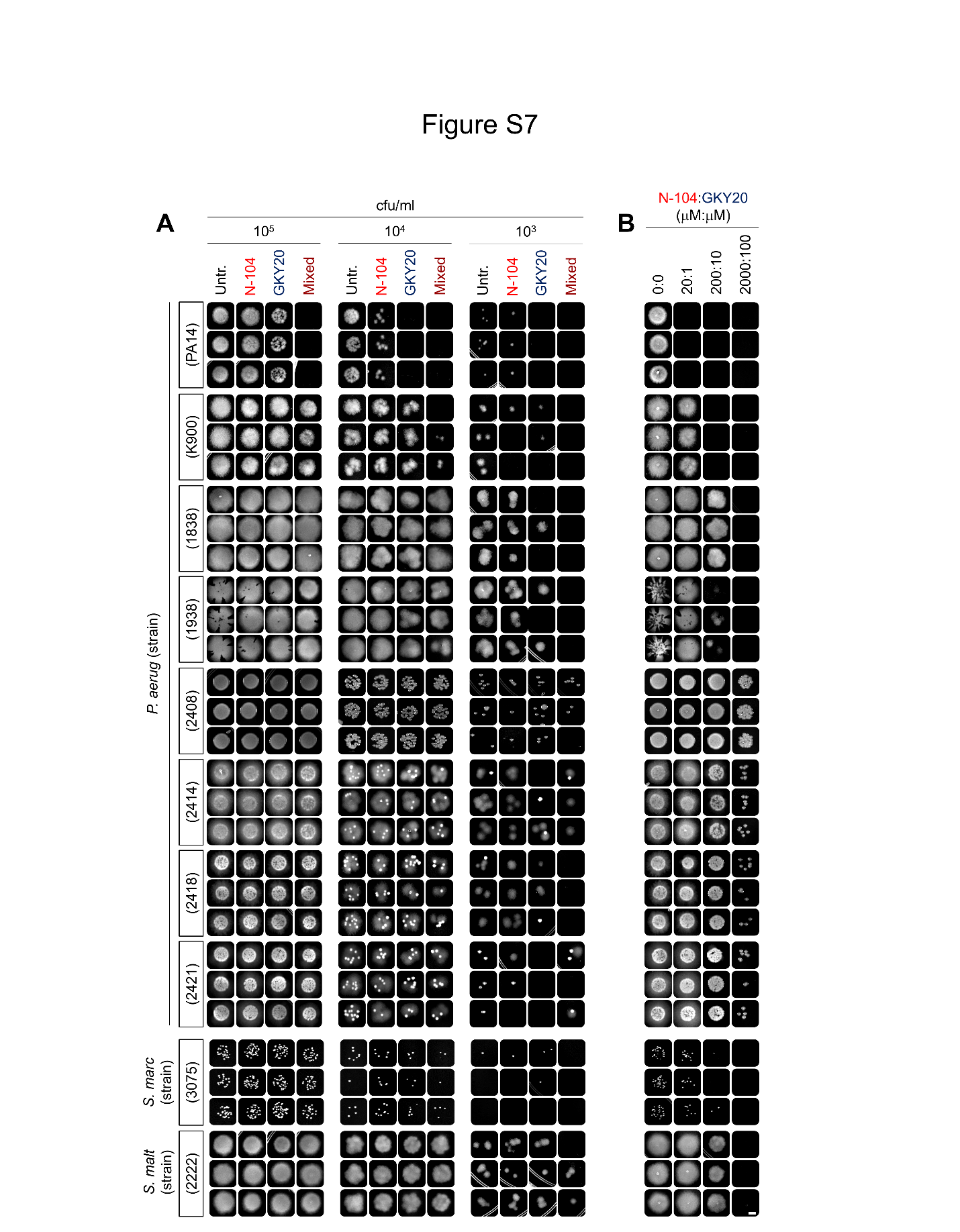


Supplementary Fig. 7. N-104 and N-104 plus GKY20 killing assays of clinical isolates versus PA14 as biological replicates related to Fig. 7. *A*, CFU replicate optimization assays addressing the appropriate cfu/ml of clinical isolate overnight cultures. Each human clinical isolate (10^3^, 10^4^ or 10^5^ cfu/ml) or PA14 was incubated in suspension for 8 hrs without (‘Untr.’) or with 20:1 N-104:GKY20 (μM:μM) in 300 mOsm/l phosphate buffer, and then plated onto LB agar. *B*, Biological replicates of Fig. 7A CFU assay in which overnight cultures of each human clinical isolate or of PA14 (10^5^ cfu/ml) was incubated in suspension for 8 hrs with 0, 20:1, 200:10 or 2000:100 N-104:GKY20 (µM:µM) in 300 mOsm/l phosphate buffer and then plated onto LB agar. Scale bar is 2 mm.

**Resources Table**

| **REAGENT or RESOURCE** | **SOURCE** | **IDENTIFIER** |
| --- | --- | --- |
| Antibodies | | |
| Rabbit polyclonal anti-His-tag | Cell Signaling Technology, Inc | Cat #: 2365S,  RRID:AB_2115720 |
| Rabbit polyclonal anti-lacritin C-terminus | DOI: 10.1167/iovs.12-11488 | RRID:AB_3094921 |
| Rabbit polyclonal anti-lacritin N-terminus | DOI: 10.1167/iovs.11-8729 | RRID:AB_3094920 |
| Rabbit anti-coagulation factor II/thrombin | Novus Biologicals | Cat #: NBP1-58268,  RRID: AB_11023777 |
| IRDye® 680RD donkey anti-rabbit IgG 2^o^ antibody | LI-COR | Cat #: 926-68073,  RRID:AB_10954442 |
| Bacterial strains | | |
| *Escherichia coli* K12 | DOI: 10.1038/msb4100050 | Wild-type strain K12 |
| *Escherichia coli* Keio collection |  | Keio collection, Kan^r (a)^ |
| *Escherichia coli* BL21 (DE3) | BioLabs Inc. | Cat #: C2527I, Chl^r (a)^ |
| *Escherichia coli* OP50 | Caenorhabditis Genetics Center | Wild-type strain, Str^r (a)^ |
| *Pseudomonas aeruginosa* PA14 | DOI: 10.1073/pnas.0511100103 | Wild-type strain PA14, Amp^r^, Kan^r^, Mem^r (a)^ |
| *Pseudomonas aeruginosa* PA14 Δ*feoB* |  | *feoB* transposon mutant, Gen^r (a)^ |
| *Pseudomonas aeruginosa* PA14 Δ*spuG* |  | *spuG* transposon mutant, Gen^r (a)^ |
| *Pseudomonas aeruginosa* PA14 Δ*yaiW* |  | *yaiW* transposon mutant, Gen^r (a)^ |
| *Pseudomonas aeruginosa* K900 | University of Pittsburgh's Charles T. Campbell Ophthalmic Microbiology Laboratory | Clinical isolate; Stain K900, Bac^r^, Van^r^, Cef^r^, Sulf^r (a)^ |
| *Pseudomonas aeruginosa* K1838 |  | Clinical isolate; Strain K1838, Bac^r^, Van^r^, Cef^r^, Sulf^r (a)^ |
| *Pseudomonas aeruginosa* K1938 |  | Clinical isolate; Strain K1938; Bac^r^, Van^r^, Cef^r^, Sulf^r (a)^ |
| *Pseudomonas aeruginosa* K2408 |  | Clinical isolate; Strain K2408; Bac^r^, Van^r^, Cef^r^, Sulf^r (a)^ |
| *Pseudomonas aeruginosa* K2414 |  | Clinical isolate; Strain K2414; Bac^r^, Van^r^, Cef^r^, Sulf^r^, Ofl^r^, Mox^r (a)^ |
| *Pseudomonas aeruginosa* K2418 |  | Clinical isolate; Strain K2418; Bac^r^, Van^r^, Cef^r^, Sulf^r (a)^ |
| *Pseudomonas aeruginosa* K2421 |  | Clinical isolate; Strain K2421, Bac^r^, Van^r^, Cef^r^, Sulf^r (a)^ |
| *Serratia marcescens* K3075 |  | Clinical isolate; Strain K3075; Bac^r^, Van^r^, Cef^r^, Sulf^r^, PB^r (a)^ |
| *Stenotrophomonas maltophilia* K2222 |  | Clinical isolate; Strain K2222; Bac^r^, Van^r^, Cef^r^, Gen^r^, Tob^r (a)^ |
| Biological samples | | |
| Basal tears from normal human eyes | This paper | N/A |
| Synthetic peptides (GenScript, Piscataway NJ) and chemicals | | |
| N-104 (AQKLLKKFSLLKPWA) | This paper | N/A |
| K107S (AQSLLKKFSLLKPWA) | This paper | N/A |
| L108S (AQKSLKKFSLLKPWA) | This paper | N/A |
| L109S (AQKLSKKFSLLKPWA) | This paper | N/A |
| K110S (AQKLLSKFSLLKPWA) | This paper | N/A |
| K111S (AQKLLKSFSLLKPWA) | This paper | N/A |
| F112S (AQKLLKKSSLLKPWA) | This paper | N/A |
| L114S (AQKLLKKFSSLKPWA) | This paper | N/A |
| L115S (AQKLLKKFSLSKPWA) | This paper | N/A |
| K116S (AQKLLKKFSLLSPWA) | This paper | N/A |
| W118S (AQKLLKKFSLLKPSA) | This paper | N/A |
| LFW:S (AQKSSKKSSSSKPSA) | This paper | N/A |
| Cys(N)-N-104 (CAQKLLKKFSLLKPWA) | This paper | N/A |
| FITC-N-104 (FITC-Ahx-AQKLLKKFSLLKPWA) | This paper | N/A |
| JanFl-N-104 (JaneliaFluor-CAQKLLKKFSLLKPWA) | This paper | N/A |
| Chloroalkane-N-104 (Cl-{C11H21O3N}-N-104) | This paper | N/A |
| C-95 (EDASSDSTGADPAQEAGTSKPNEE) | This paper | N/A |
| Cys(N)-C-95 (CEDASSDSTGADPAQEAGTSKPNEE) | This paper | N/A |
| FITC-C-95 (FITC-Ahx-EDASSDSTGADPAQEAGTSKPNEE) | This paper | N/A |
| JanFl-C-95 (JaneliaFluor-CEDASSDSTGADPAQEAGTSKPNEE) | This paper | N/A |
| Chloroalkane-C-95 (Cl-{C11H21O3N}-C-95) | This paper | N/A |
| N-80/C-25 (AKAGKGMHGGVPGG) | This paper | N/A |
| GKY20 (GKYGFYTHVFRLKKWIQKVI) | This paper | N/A |
| GSN (gelsolin) (QRLFQVKGRR) | This paper | N/A |
| CST4 (cystatin S) (SSSKEENRIIPGGI) | This paper | N/A |
| Syndecan-1 pep19-30 (DNFSGSGAGAL) | This paper | N/A |
| FeoB OL1 peptide (INIGGALQP) | This paper | N/A |
| FeoB OL2 peptide (LEDSGYMARAAFVMDRLMQ) | This paper | N/A |
| FeoB OL3 peptide (GAFFGQGGA) | This paper | N/A |
| FeoB OL5 peptide (ATFAA) | This paper | N/A |
| PotH OL2 peptide (WMGILKNNGVLNNFLLWLGVIDQPLTILHTN) | This paper | N/A |
| PotH OL3 peptide (ELLGGPDSIMIGRVLWQEFFNNRDW) | This paper | N/A |
| Acetonitrile | Sigma-Aldrich | Cat #: 34998 |
| Alamar Blue | Invitrogen | Cat #: DAL1100 |
| CHAPS detergent | Fisher Scientific | Cat #: 28299 |
| α-Cyano-4-hydroxycinnamic acid | Sigma-Aldrich | Cat #: 70990 |
| Diethylpyrocarbonate | Fisher Scientific | Cat #: AC170250250 |
| Dithiolthreitol, DTT | Fisher Scientific | Cat #: BP172-25 |
| Dodecyl-β-D-maltopyranoside | Alfa Aesar | Cat #: J66869 |
| Ferrozine | Sigma-Aldrich | Cat #: 160601 |
| Formaldehyde | Polysciences Inc. | Cat #: 18814 |
| JaneliaFluor 549 Maleimide | TOCRIS | Cat #: 6500 |
| lauryl maltose neopentyl glycol | Fisher Scientific | Cat #: A50940 |
| Lysozyme | Fisher Scientific | Cat #: PI89833 |
| Neocuproine | Sigma-Aldrich | Cat #: N1501 |
| Ni-sepharose^TM^ | GE Healthcare | Cat #: 17-3712-01 |
| n-octyl-β-D-glucopyranoside | Sigma-Aldrich | Cat #: O8001 |
| OptiPhase HiSafe 3 scintillation fluid | PerkinElmer | Part #: 1200.437 |
| 1-palmitoyl-2-oleoyl-sn-glycero-3-phosphocholine | Avanti Polar Lipids | Cat #: 850457P |
| 1-palmitoyl-2-oleoyl-sn-glycero-3-phospho-(1'-rac-glycerol) | Avanti Polar Lipids | Cat #: 840457 |
| 1-palmitoyl-2-oleoyl-sn-glycero-3-phosphoethanolamine | Avanti Polar Lipids | Cat #: 850757 |
| Pierce^TM^ Control Agarose Resin | Fisher Scientific | Cat #: 26150 |
| Proteinase K | Fisher Scientific | Cat #: EO0491 |
| [1,4-^14^C]putrescine | American Radiolabeled Chemicals | Cat #: ARC-0245-250 |
| Putrescine | MP Biomedicals | Cat #: 100450 |
| Rhodamine chloroalkane | Promega® | Cat #: G3221 |
| RNaseZap^TM^ | Invitrogen | Cat #: AM9780, AM9782 |
| RNA-protect Bacteria Reagent | Qiagen | Cat #: 76506 |
| Sephadex^TM^ G-10 | Cytiva | Cat #: 17001001 |
| [1,4-^14^C]spermidine | American Radiolabeled Chemicals | Cat #: ARC-3138-50 |
| Spermidine | Acros organics | Cat #: 132740010 |
| SulfoLink® Coupling Resin | Fisher Scientific | Cat #: 20401 |
| SYTOX orange | Fisher Scientific | Cat #: S11368 |
| Trifluoroacetic acid | Chem-Impex International | Cat #: 02883 |
| Trypsin EDTA | Gibco | Cat #: 25200-056 |
| Critical commercial assays | | |
| EndoFree® Plasmid Maxi Kit | Qiagen | Cat #: 12362 |
| RNeasy mini kit | Qiagen | Cat #: 74104 |
| NEBNext® rRNA Depletion Kit (Bacteria) | New England BioLabs | Cat #: E7850L |
| BCA protein assay | bioWORLD | Cat #: 20831001-1 |
| Deposited data | | |
| RNA-seq data | NCBI GEO data set | Accession ID:  GSE253123 |
| Tear proteomics | MassIVE data set | Accession ID:  MSV000094085 |
| PA14 proteomics | MassIVE data set | Accession ID:  MSV000094086 |
| Experimental models: Cell lines | | |
| Sheep red blood cells | MP Biomedicals | Cat #: 0855876 |
| Experimental models: Organisms/strains | | |
| *Caenorhabditis elegans*: Wild-type strain N2 | Caenorhabditis Genetics Center | Wild-type strain N2,  RRID:WB-STRAIN:WBStrain00000003 |
| Mouse | The Jackson Laboratory | C57BL/6 (female, 7 weeks old), RRID:MGI:2159769 |
| Oligonucleotides | | |
| Primers for site-directed mutagenesis experiments, see Table S4 | This paper | N/A |
| Recombinant DNA | | |
| *feoB* | DOI: 10.1016/j.pep.2014.06.012 | Addgene ID: 216753  (*P. aeruginosa*’s *feoB* in pET41-a(+) vector with T7 promoter & *lac*-operator system, C-term. 8xHis, Kan^r (a)^) |
| *feoB* OL1 deletion (INIGGALQP) | This paper | Addgene ID: 216741 |
| *feoB* OL2 deletion (LEDSGYMARAAFVMDRLMQ) | This paper | Addgene ID: 216742 |
| *feoB* OL3 deletion (GAFFGQGGA) | This paper | Addgene ID: 216743 |
| *feoB* OL4 deletion (131 residues) | This paper | Addgene ID: 216744 |
| *feoB* OL5 deletion (ATFAA) | This paper | Addgene ID: 216745 |
| *feoB* OL2/4 substitution | This paper | Addgene ID: 216746 |
| *feoB* OL3/4 substitution | This paper | Addgene ID: 216747 |
| *feoB* OL5/4 substitution | This paper | Addgene ID: 216748 |
| *potH* | DNASU Plasmid Repository (https://dnasu.org/DNASU/Home.do) | ID: EcCD00397460  (*E. coli*’s *potH* in PCDF Bravo vector with T7 promoter & *lac*-operator system, C-term. 10xHis, Str^r (a)^) |
| *potH* OL1 deletion (FKISLAEMARAIPPYTELMEWADGQLSITLNLGNFLQLTDDPLYFDAYLQSLQ) | This paper | Addgene ID: 216749 |
| *potH* OL2 deletion (WMGILKNNGVLNNFLLWLGVIDQPLTILHTN) | This paper | Addgene ID: 216750 |
| *potH* OL3 deletion (ELLGGPDSIMIGRVLWQEFFNNRDW) | This paper | Addgene ID: 216751 |
| *potH* OL3/2 substitution | This paper | Addgene ID: 216752 |
| *cyto-Halo* | DOI: 10.1021/acsinfecdis.2c00435 | HaloTag in pET21(b)+ vector with T7 promoter & lac-operator system, Amp^r (a)^ |
| *peri-Halo* |  | Cyto_Halo containing an amine terminal recognition sequence in pET21(b)+ vector with T7 promoter & lac-operator system, Amp^r (a)^ |
| Software and algorithms | | |
| Attune cytometric software (version 5.3.0) | Thermo Fisher Scientific | https://www.thermofisher.com |
| DESeq2 (version 1.38.3) | Bioconductor | https://support.bioconductor.org |
| GraphPad Prism (version 10.2.0) |  |  |
| IDEAS® program (version 6.2) | Amnis® system | https://www.emdmillipore.com/US/en/20150122_174404 |
| ImageJ (version 1.48v) | National Institute of Health | https://imagej.net/ij/docs/install/windows.html |
| Image Studio^TM^ Lite (version 5.2.5) | LI-COR | https://www.licor.com/bio/image-studio-lite/ |
| NEBase Changer (version 2.4.3) | New England Biolabs Inc. | https://nebasechanger.neb.com/?# |
| SnapGene Viewer (version 5.0.4) | SnapGene | https://www.snapgene.com/support/downloads |
| Other | | |
| Acetic acid | Fisher Scientific | Cat #: A38SI-212 |
| Ammonium acetate | Fisher Scientific | Cat #: A639 |
| Ampicillin | Fisher Scientific | Cat #: BP1760-5 |
| Ascorbic acid | Sigma-Aldrich | Cat #: A4544 |
| 2,2’-bipyridyl | Sigma-Aldrich | Cat #: D216305 |
| L-cysteine | Alfa Aesar | Cat #: A10435 |
| Dimethyl sulfoxide | Fisher Scientific | Cat #: D128-500 |
| FeSO_4_ | Sigma-Aldrich | Cat #: 215422 |
| Fish gelatin | Sigma-Aldrich | Cat #: G7765 |
| Formic acid | Fisher Scientific | Cat #: A117-50 |
| Gentamicin | Alfa Aesar | Cat #: J62834 |
| Imidazole | Fisher Scientific | Cat #: 122025000 |
| Isopropyl-β-D-1-thiogalactopyrannoside | Fisher Scientific | Cat #: R0392 |
| Kanamycin | Fisher Scientific | Cat #: BP906-5 |
| Laemmli buffer (4x) | Bio-Rad | Cat #: 1610737 |
| LB Broth medium | Fisher Scientific | Cat #: BP9723-2 |
| Luria-Bertani agar | Fisher Scientific | Cat #: BP1425-2 |
| M9 minimal salts | Gibco | Cat #: A1374401 |
| β-mercaptoethanol | Sigma-Aldrich | Cat #: M7522 |
| Mini-PROTEIN TGX Gel | Bio-Rad | Cat #: 4561094 |
| MWCO (50 kDa) | Amicon® | Cat #: UFC505024 |
| MWCO (30 kDa) | Amicon® | Cat #: UFC503024 |
| MWCO (5 kDa) | Amicon® | Cat #: UFC900596 |
| Nitrocellulose membrane | GE Healthcare Life Sciences | Cat #: 10600001 |
| Protease inhibitor cocktail | cOmplete Mini | Cat #: 11836153001 |
| RDD buffer | Qiagen | Cat #: 79254 |
| Streptomycin | Sigma-Aldrich | Cat #: S650-1 |
| TE buffer | Invitrogen | Cat #: 12-090-015 |
| Terrific broth medium | Research Products International | Cat #: T15100-1000.0 |
| Triton X-100 | Bio-Rad | Cat #: 161-0407 |
| Tween-20 | Bio-Rad | Cat #: 1706531 |

Key:

(i) antibiotics (^‘a’^): Amp, ampicillin; Bac, bacitracin; Cef, cefazolin; Chl, chloramphenicol; Gen, gentamicin; Kan, kanamycin; Mem, meropenem; Mox, moxifloxacin; Ofl, ofloxacin; PB, polymyxin B; Str, streptomycin; Sulf, sulfasoxazole; Tob, tobramycin; Van, vancomycin;

(ii) antibiotic resistance (^‘r’^)

**Supplementary Table 1:** Bacterial and viral orthologs of identified N-104 mediators, as related to Fig. 2.

|  | **Protein**  **(# of residues)** ^(a)^ | ***P. aeruginosa*,**  **PA14** ^(b)^ | **Function** | **Orthologs (# of hits)** ^(c)^ |
| --- | --- | --- | --- | --- |
| Protein hits above the threshold of 0.75 (Fig. 2G) | FeoB (773) | PA14_56680 | inner membrane ferrous iron transporter | - *Gammaproteobacteria* (3814): Inclusive of *Enterobacteriaceae*, *P. aeruginosa*, *Acinetobacter baumannii* - *Streptococcus pneumoniae* (1) |
|  | PotH (317) | PA14_03950 | inner membrane polyamine transporter subunit | - *Gammaproteobacteria* (3916): Inclusive of *Enterobacteriaceae*, *P. aeruginosa*, *Acinetobacter baumannii* - *Lactobacillales* (2) |
|  | RluE (217) | PA14_12540 | GntR family member YmfC (regulatory) | - *Gammaproteobacteria* (4205): Inclusive of *Enterobacteriaceae*, *P. aeruginosa*, *Acinetobacter baumannii* - *Streptococcus pneumoniae* (1) - *Pedobacter himalayensis* (1)   Virus:   - *Escherichia phage vB_EcoM-689R6* (1) |
|  | YbaE (566) | - | periplasmic SgrR family member (regulatory) | - *Enterobacterales* (3267) |
|  | YbdM (209) | (Unnamed protein) | cytosolic ParB-like nuclease domain containing (regulatory) | - *Gammaproteobacteria* (4653): Inclusive of *Enterobacteriaceae*, *P. aeruginosa*, *Acinetobacter baumannii* - *Lactobacillales* (2) |
|  | YeeI (265) | - | zinc metallopeptidase MtfA | - *Gammaproteobacteria* (4005): Inclusive of *Enterobacteriaceae* - *Mesorhizobium sp.* (1) - *Pedobacter himalayensis* (1)   Viruses:   - Unclassified *Caudoviricetes* (5) - *Bacteriophage sp.* (1) |
|  | YhfZ (301) | - | putative transcription factor (regulatory) | - *Gammaproteobacteria* (2381): Inclusive of *Enterobacteriaceae*, *Acinetobacter baumannii* - *Fusobacteriales* (12) - *Coprothermobacter platensis* (1) |
|  | YjhQ (181) | - | growth promoting antitoxin | - *Gammaproteobacteria* (1010): Inclusive of *Enterobacteriaceae* - *Thermodesulfobacteriota* (102)   Archaea:   - *Stenosarchaea group* (41) - *Candidatus Bathyarchaeota archaeon* (18) - *DPANN group* (2)   Symbiodinium:   - *Symbiodinium sp. CCMP2592* (1) - *Symbiodinium microadriaticum* (2) |
|  | YobD (152) | - | conserved inner membrane  protein | - *Gammaproteobacteria* (4430): Inclusive of *Enterobacteriaceae*, *Acinetobacter baumannii* |
|  | WcaD (405) | - | inner membrane colanic acid polymerase | - *Gammaproteobacteria* (3878): Inclusive of *Enterobacteriaceae*, *P. aeruginosa* - *Streptococcus pneumoniae* (1) - *Pedobacter himalayensis* (1) |
|  | | | | |
| Below the threshold (Fig. 2D) | TauD (283) | TauD | taurine dioxygenase | - *Gammaproteobacteria* (2934): Inclusive of *Enterobacteriaceae*, *P. aeruginosa*, *Acinetobacter baumannii* |
|  | YaiW (364) | PA14_29270 | surface-exposed  outer-membrane lipoprotein | - *Gammaproteobacteria* (3871): Inclusive of *Enterobacteriaceae*, *P. aeruginosa*, *Acinetobacter baumannii* |

^(a)^ *https://shigen.nig.ac.jp/ecoli/pec_w3110/*

^(b)^ The ‘gene ID (gene name)’⏐‘# of residues’⏐‘identity’ (*http://pa14.mgh.harvard.edu/cgi-bin/pa14/search.cgi*):

PA14_56680 (*feoB*)⏐766⏐66%; PA14_03950 (*spuG*)⏐293⏐65%; PA14_12540⏐189⏐65%;

Unnamed protein⏐211⏐52% : NCBI Ref. Seq.: WP_003088853.1; *tauD*⏐277⏐60%; PA14_29270 (*yaiW*)⏐367⏐63%

BLAST parameters: Data base: ‘Non-redundant protein sequences’⏐Included: *Pseudomonas aeruginosa* UCBPP-PA14 (taxid:208963)⏐Max target sequences: 1000⏐Expect threshold: 0.1.

^(c)^ BLAST parameters: Data base: ‘Non-redundant protein sequences’⏐Excluded: *E. coli* (taxid:562)⏐Max target sequences: 1000⏐Expect threshold: 0.001.

**
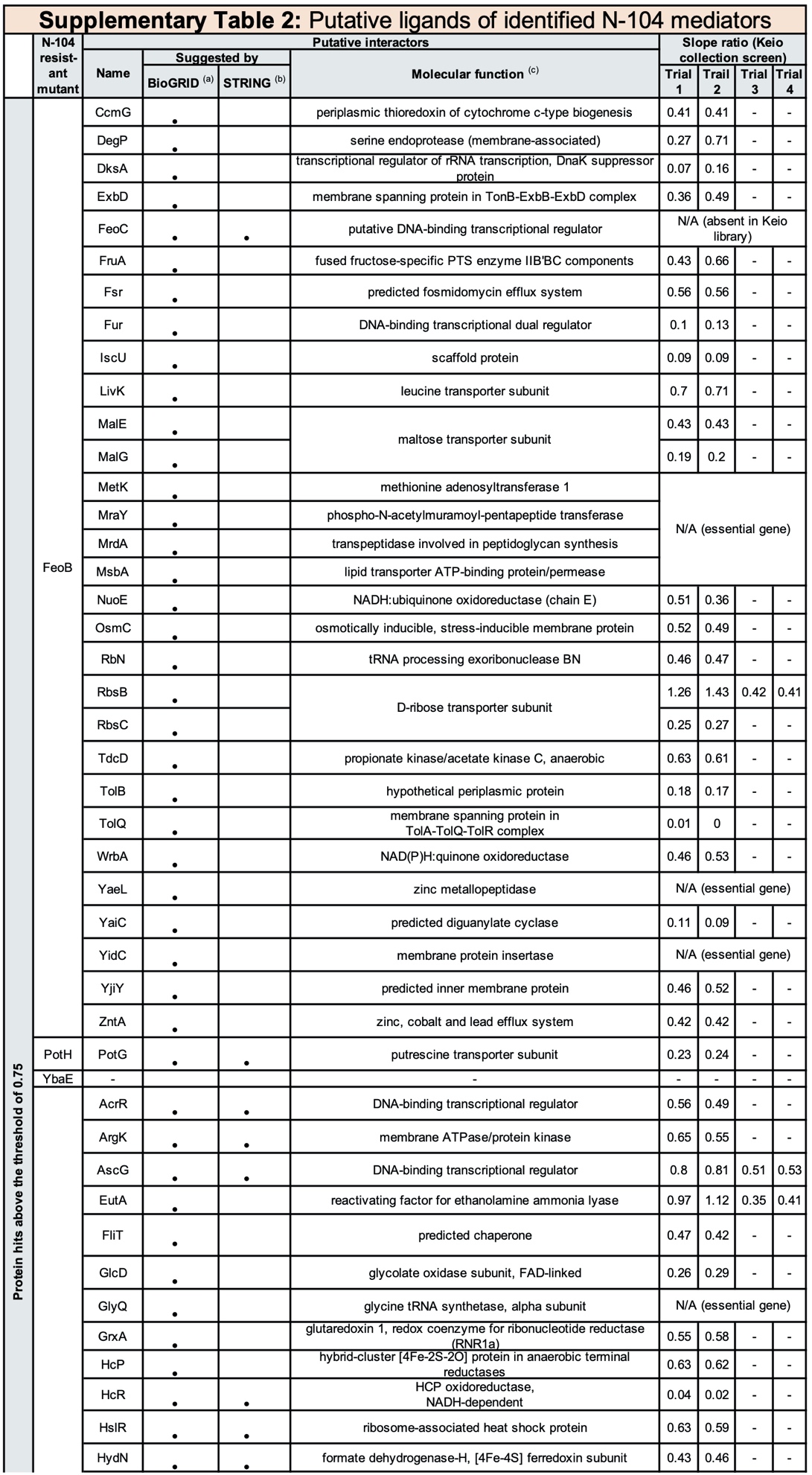
**

**
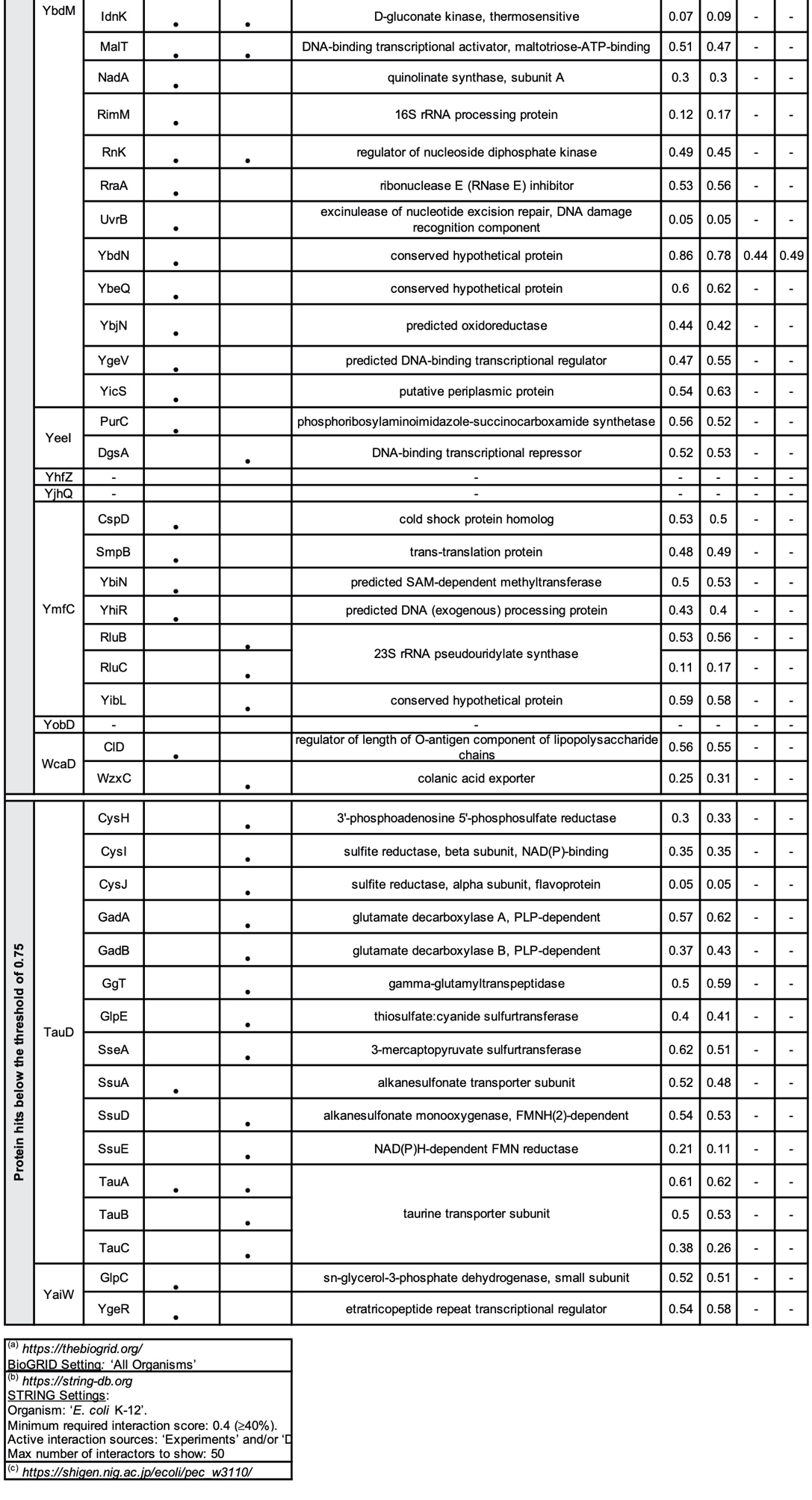
**

**Supplementary Table 3:** Imaging flow cytometry compliant items, as related to Fig. 3.

| **Item** | **Information** |
| --- | --- |
| Purpose | Imaging flow cytometry of *P. aeruginosa* PA14 cells one hour after treatment with FITC-N-104 (or -C-95) |
| Keywords | Cellular accumulation, lacritin peptides, *P. aeruginosa* PA14 |
| Experiment variables | Peptide structure and concentration |
| Organization | Flow Cytometry Core Facility at University of Virginia, Charlottesville, VA, USA |
| Primary contact | Michael D. Solga (mds4z@virginia.edu) |
| Date/time period of experiment | August 2021 – March 2022 |
| Conclusions | In contrast to FITC-C-95, substantial intracellular FITC-N-104 fluorescence was apparent at either 100 or 10 μM concentrations |
| Quality control measures | Positive control using ethanol permeabilization |
| Biological sample source | DOI: 10.1073/pnas.0511100103 |
| Biological organism | *P. aeruginosa* PA14 strain. |
| Sample treatment | 0.5 ml of 10^6^ cfu/ml PA14 cells in 1 mM Na_2_HPO_4_, 0.18 mM KH_2_PO_4_, 0.27 mM KCl, 13.7 mM NaCl, pH 7.2 were incubated aerobically for one hour at 35°C with 10 or 100 μM FITC-N-104 (without, or as positive control with 70% ethanol) or with negative control FITC-C-95. Cells were subsequently washed in the same buffer, pelleted, treated with 100 μl of 0.25% trypsin EDTA (Gibco, 25200-056) for 5 min at 35°C to remove surface attached peptide, further washed and pelleted, resuspended in 100 μl in the same buffer for fixation with an equal volume of 8% formaldehyde (18814, Polysciences Inc.). |
| Fluorescence reagents | FITC-Ahx-AQKLLKKFSLLKPWA  FITC-Ahx-EDASSDSTGADPAQEAGTSKPNEE |
| Instrument manufacturer | CYTEK |
| Instrument model | Amnis Imagestream X Mark II imaging flow cytometer |
| Instrument configuration  and settings | - Laser/Power: 405 nm/120 mW; 488 nm/200 mW (channel 2; FITC channel); 560 nm/200 mW; 658 nm/150 mW - Bandpass filters: 480-560 nm (for 488 nm laser); 740-800 nm (for dark-field side scattering) - 60X lens with numerical aperture of 0.9 - Field-of-view: 40x170 μm |
| Compensation | Performed on IDEAS version 6.2 program (Amnis, Seattle WA) |
| Data transformation | The fluorescence intensity were analyzed using IDEAS version 6.2 program (Amnis, Seattle WA) |
| Gate description, statistics,  and boundaries | 500-1000 counts were analyzed per replicate. To ensure the exclusion of debris and out-of-focus cells, 'Gradient-RMS' ('average slope spanning three pixels in an image'; 0-100) versus Area ('number of pixels in an image reported in square microns; 1-200) of channel 1 (bright-field) was plotted for each experiment with over 85% of the data falling within the ‘cells-in-focus’ gate. The fluorescence intensity values of FITC-positive cells (Intensity-MC, channel 2) were plotted as histograms with a bin number of 100. FITC-positive cells were selected based on histograms generated from the positive control sample (≥ 0.002). |


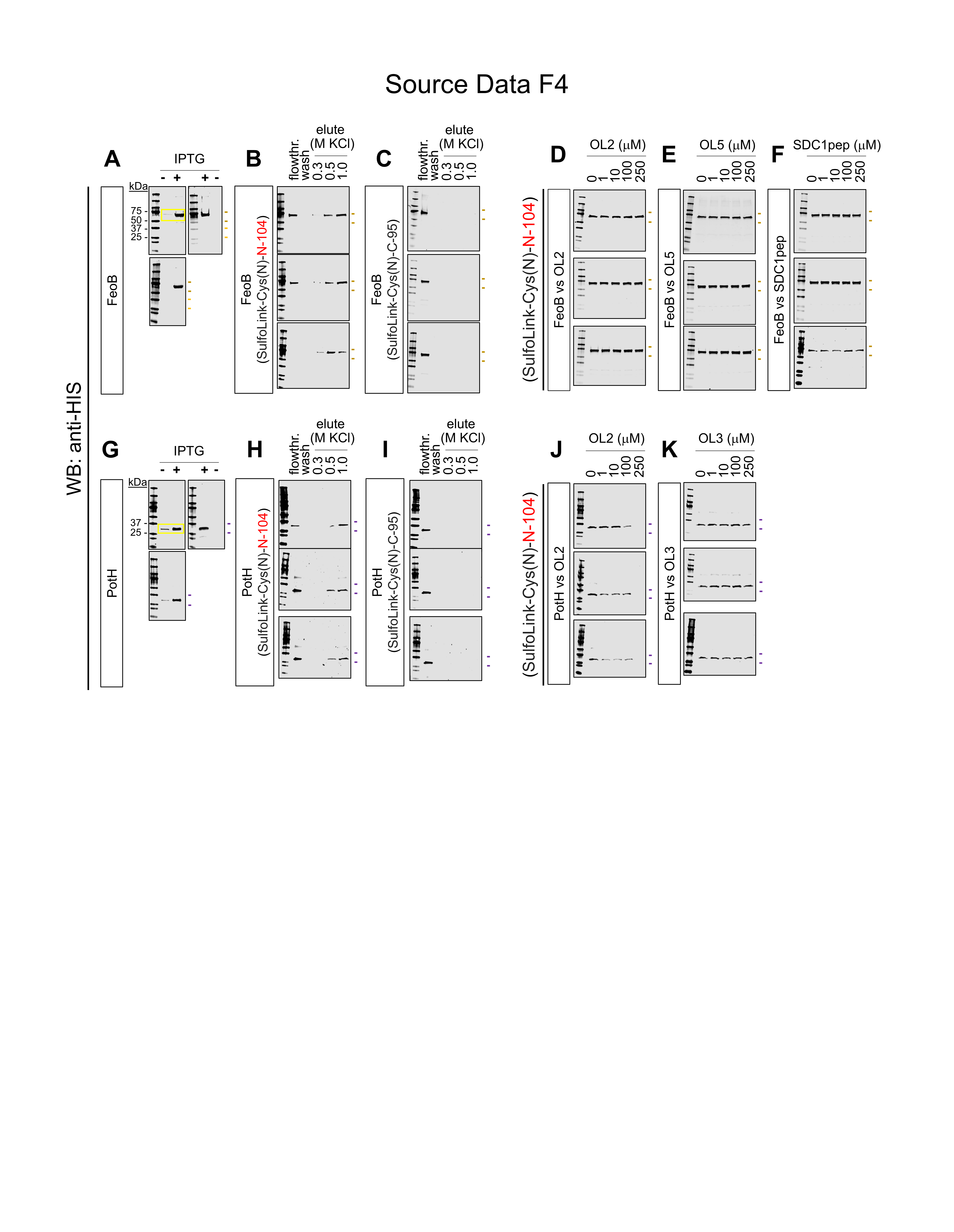


Source Data F4. **Inner membrane Fe^2+^ and polyamine transporters FeoB and PotH respectively bind N-104.** *A*, Recombinant expression of his tagged FeoB. *B,* His-tagged FeoB binding assay with SulfoLinkCys(N)-N-104 columns. Overnight cultures of *E. coli* Lemo21 (DE3) cells overexpressing His-tagged FeoB were lysed in buffered 2% dodecyl-β-D-maltopyranoside with protease inhibitors, captured on nickel columns, eluted, subjected to a buffer change and then passed onto a SulfoLinkCys(N)-N-104 column at 4ºC. Shown is the flowthrough (flowthr), last wash fraction and 0.3 – 1 M KCl elution fractions as detected by anti-His Western blotting. *C*, The same procedure as in *B* in which lysate was passed over negative control SulfoLinkCys(N)-C-95 columns. *D,* The same procedure was performed in *B*, but in the presence of increasing concentrations of outer loop 2 (OL2) synthetic peptide. *E,* The same procedure was performed in *B*, but in the presence of increasing concentrations of outer loop 5 (OL5) synthetic peptide. *F,* The same procedure was performed in *B*, but in the presence of increasing concentrations of SDC1 synthetic peptide. *G*, Recombinant expression of his tagged PotH. *H,* His-tagged PotH binding assay with SulfoLinkCys(N)-N-104 columns. Overnight cultures of PA14 overexpressing His-tagged PotH were lysed in buffered 2% dodecyl-β-D-maltopyranoside with protease inhibitors, captured on nickel columns, eluted, subjected to a buffer change and then passed onto a SulfoLinkCys(N)-N-104 column at 4ºC. Shown is the flowthrough (flowthr), last wash fraction and 0.3 – 1 M KCl elution fractions as detected by anti-His Western blotting. *I,* The same procedure was performed as in *H*, but with negative control SulfoLinkCys(N)-C-95 columns. *J*, The same procedure as in I, but in the presence of increasing concentrations of outer loop synthetic peptide 2 (OL2). *K*, The same procedure as in *J*, but in the presence of increasing concentrations of outer loop synthetic peptide 3 (OL3).

^
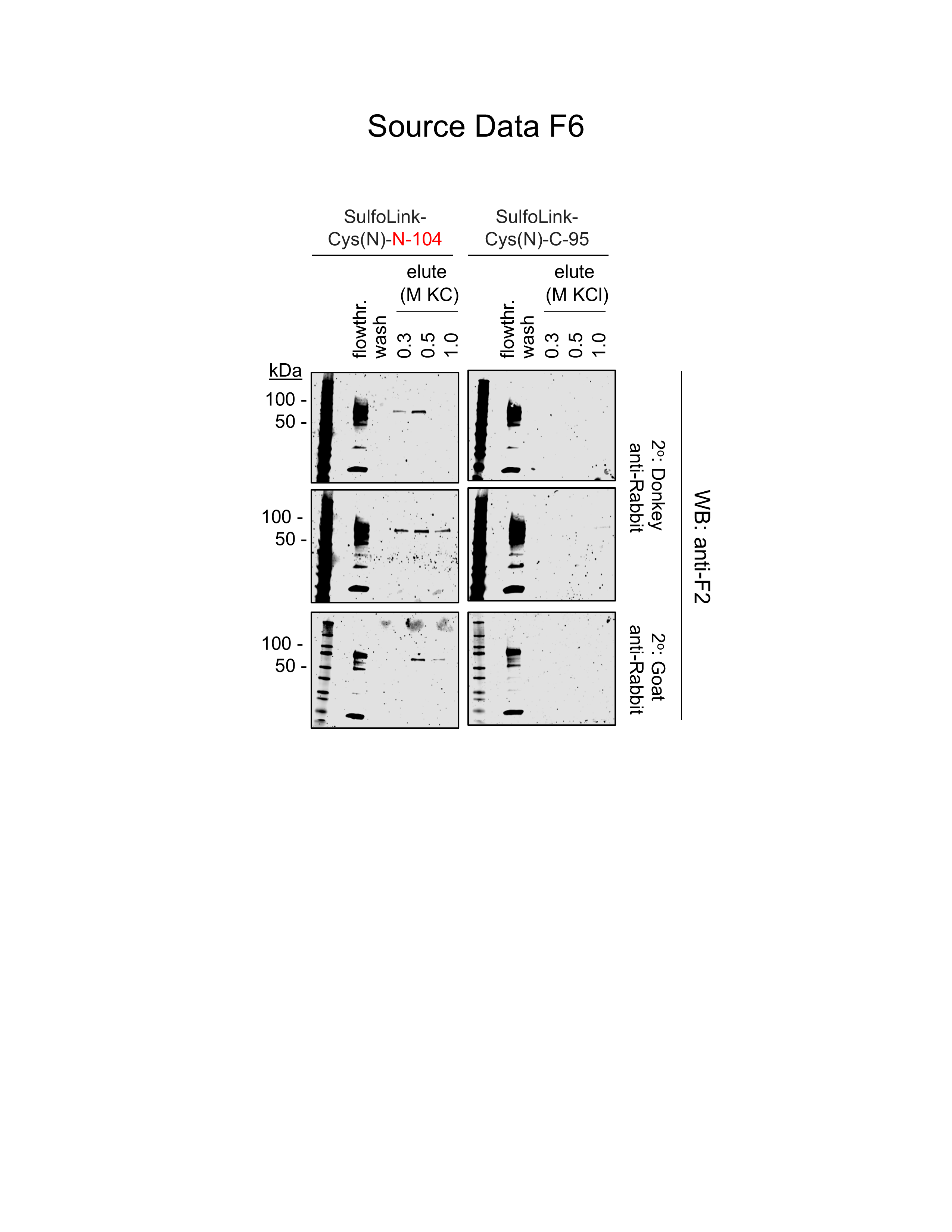
^

Source Data F6. **N-104 protein affinity screen of antimicrobial-rich human tears captures thrombin: thrombin GKY20 antimicrobial synergy with N-104.**

Immunodection of F2 (thrombin) in the 0.5 M KCl eluant off SulfoLinkCys(N)-N-104 but not SulfoLinkCys(N)-C-95 columns. Equal volumes of the flow through (flowthr), final wash fraction and KCl elution fractions from *B* were subjected to 4 - 20% SDS-PAGE without sample boiling for anti-F2 immunodetection using LI-COR.
